# Next generation histology methods for three-dimensional imaging of fresh and archival human brain tissues

**DOI:** 10.1038/s41467-018-03359-w

**Published:** 2018-03-14

**Authors:** Hei Ming Lai, Alan King Lun Liu, Harry Ho Man Ng, Marc H. Goldfinger, Tsz Wing Chau, John DeFelice, Bension S. Tilley, Wai Man Wong, Wutian Wu, Steve M. Gentleman

**Affiliations:** 1 School of Biomedical Sciences, Li Ka Shing Faculty of Medicine, The University of Hong Kong, Hong Kong, 852 Hong Kong; 2 Neuropathology Unit, Division of Brain Sciences, Imperial College London, Burlington Danes Building, Hammersmith Campus, Du Cane Road, London, W12 0NN UK; 3 GMH Institute of CNS Regeneration, Jinan University, 601 Huangpu Avenue West, Guangzhou, 510632 China; 4 Re-Stem Biotechnology Co., Ltd, 2588 Wuzhong Road, Suzhou, 215310 China

## Abstract

Modern clearing techniques for the three-dimensional (3D) visualisation of neural tissue microstructure have been very effective when used on rodent brain but very few studies have utilised them on human brain material, mainly due to the inherent difficulties in processing post-mortem tissue. Here we develop a tissue clearing solution, OPTIClear, optimised for fresh and archival human brain tissue, including formalin-fixed paraffin-embedded material. In light of practical challenges with immunostaining in tissue clearing, we adapt the use of cresyl violet for visualisation of neurons in cleared tissue, with the potential for 3D quantification in regions of interest. Furthermore, we use lipophilic tracers for tracing of neuronal processes in post-mortem tissue, enabling the study of the morphology of human dendritic spines in 3D. The development of these different strategies for human tissue clearing has wide applicability and, we hope, will provide a baseline for further technique development.

## Abstract (web summary)

Current available tissue clearing techniques are mostly used for rodent tissues. Here, the authors develop OPTIClear solution for fresh and archival human brain tissue clearing and establish associated protocols for three-dimensional histological investigations.

## Introduction

The brain is arguably the most complex organ in the human body. For centuries, efforts have been made to try and understand the structural and functional connections within the brain. At the macroscopic level, diffusion tensor imaging and functional magnetic resonance imaging are beginning to unravel some of the complex connections between different anatomical regions. However, due to the spatial resolution of these imaging modalities, they lack the ability to reveal details at the microcircuit or cellular level^1^.

As the founder of the neuron dogma Santiago Ramόn y Cajal stated, “the key to understanding turns on the nature of available instrumentation”^2^. A recent resurgence of interest in tissue clearing techniques that can render opaque tissue transparent, combined with the advances in fluorescent labelling probes and imaging technologies, has proven useful in the study of neuropathology in three dimensions (3D). For example, CLARITY^3^, which renders a piece of acrylamide hydrogel-hybridised tissue transparent by detergent-based delipidation, has been used to study Alzheimer’s^4^, Lewy body^5^ and neurodevelopmental^3,6^ pathologies in 3D. Furthermore, ScaleS, a tissue-clearing strategy using serial immersion of tissue in a sorbitol-based solution, successfully demonstrated the spatial relationships between microglia and amyloid-beta plaques in Alzheimer’s disease (AD) brain tissues^7^. Despite the great potential of tissue clearing in human neuropathological research, these techniques were initially developed for rodent brain research. Although attempts have been made to simplify and improve efficiency of tissue clearing in human tissues^8^, there are many unresolved issues that remain to be addressed. In particular, the limited penetration of antibodies appears to be a universal problem faced by many groups working on tissue clearing^9^. Hence, it is important to explore whether more traditional histological staining strategies can be used effectively with modern tissue clearing methods.

It is increasingly apparent that the inherent differences between rodent and human tissue warrants the development of a tissue-clearing method dedicated to human brain tissue^5^. Fundamentally, there are significant differences in gross size, physiochemical properties and neuronal and myelin densities between rodent and primate brains^10^, making clearing more challenging in human tissues. Furthermore, although the study of neuronal connectivity can be aided by fluorescent labelling technologies using genetic or viral tracers^11^, such techniques are not available for human post-mortem studies. From a practical point of view, working with human tissue is further complicated by uncontrollable variables including the pre-mortem agonal state of the patient and the post-mortem delay.

On the basis of our understanding of the mechanisms of tissue clearing and the properties of human neural tissues, we have deconstructed some of the current techniques and identified three major aims for this study: (1) to develop a refractive index homogenisation solution optimised for human tissue clearing research; (2) to optimise tissue clearing for prolonged formalin-fixed and paraffin-embedded materials, thereby freeing up archival tissues for research; and (3) to explore the compatibility of traditional non-immunohistochemical histology staining methods with tissue clearing.

Due to the inherent variability of human tissue, no single protocol is appropriate in all situations. Our objective here was to develop a series of strategies for human brain tissue clearing which are simple to execute and can be used as the basis for further 3D histological studies. Here we present the development of a refractive index homogenisation reagent, OPTIClear (for Optical Properties-adjusting Tissue-Clearing agent), which is optimised for tissue clearing in both fresh and archival human brain tissues, including formalin-fixed paraffin-embedded (FFPE) materials. This has enabled us to establish various protocols for 3D histological investigations for the study of the human brain in health and disease.

## Results

### Development of OPTIClear

Refractive index homogenisation is a crucial step in tissue clearing. It was previously suggested by Richardson and Lichtman^12^ that the role of refractive index-matching solutions is to reduce inhomogeneity within tissues to minimise scattering of light. We further hypothesised that tissue opacity originates mostly from the refractive index mismatches between different compartments in the tissue, which is most significant between hydrophobic (e.g., lipid membranes and lipid droplets) and hydrophilic (e.g., extracellular space, cytosol and nucleus) compartments. Therefore, we reasoned that the application of compartment-selective refractive index-adjusting agents would allow matching of these compartments’ refractive indices (Supplementary Figure 1a). We performed screening experiments using a modified version of strategies by Susaki et al^13^ (Supplementary information), which led us to identify iohexol and 2,2’-thiodiethanol as appropriate refractive index-adjusting agents for hydrophilic and hydrophobic compartments, respectively (Supplementary Figure 1b). Furthermore, chemicals such as urea^7,14^ and formamide^15^ are used in many tissue-clearing agents. These are necessary for tissue clearing agents to infiltrate effectively and homogenously into the dense proteinaceous cytosol due to partial denaturation of proteins (Supplementary Figure 2) in non-delipidated tissues. However, these chemicals are strong denaturants and may destroy the tissue. To search for mild denaturants, we screened for chemicals that can solubilise boiled egg whites, and identified N-methylglucamine to replace urea in our formula. (Supplementary Information, Supplementary Figure 2). Subsequent optimisation with brain homogenates established the optimal concentrations of these three principal components (20% w/v N-methylglucamine, 25% w/v 2,2’-Thiodiethanol, 32% w/v Iohexol), and the resulting cocktail is termed OPTIClear. It is a clear colourless liquid with a refractive index of 1.47–1.48.

**Fig. 1:**
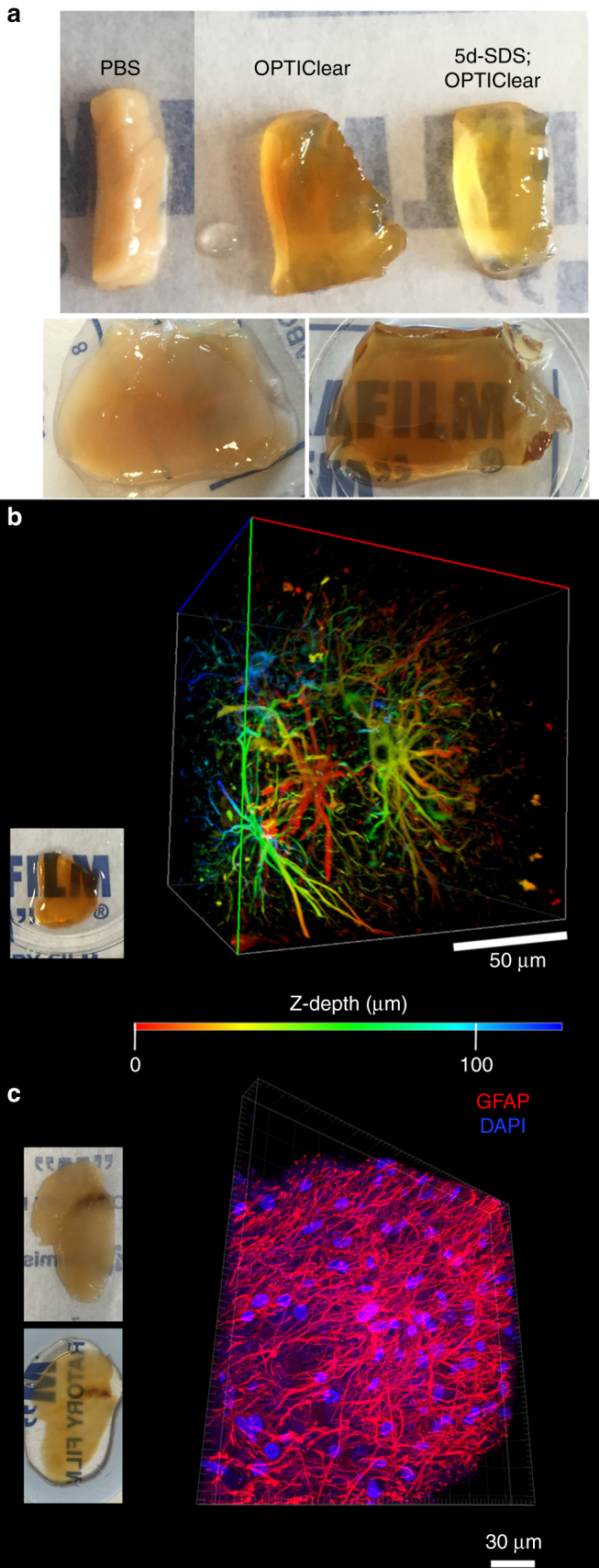
Immunofluorescence staining of archival brain tissues. **a** Upper panel: comparison of formalin-fixed human cerebellar tissue immersed in PBS (left), OPTIClear (middle) and OPTIClear after 5 days of SDS delipidation at 55 °C (right). Lower panel: comparison of 5 mm-thick, formalin-fixed human striatum tissue block after 3.5 months of SDS delipidation at 55 °C, before (left) and (after) OPTIClearing. **b** Colour depth-coded, *Z*-stack image of a block of 2 mm-thick cingulate cortex that has been formalin-fixed for 50 years, stained with antibodies against GFAP with an imaging depth of 125.57 µm. The tissue was cleared after SDS treatment for 4 months at 55 °C and cleared in OPTIClear. Scale bar = 50 µm. **c** *Z*-stack image of a piece of formalin-fixed, paraffin-embedded midbrain tissue immunostained for GFAP (red) and counterstained with DAPI (blue) after dewaxing and rehydration (*Z*-stack depth = 20.77 µm)

To test its efficacy of tissue clearing on human tissues, a 1 mm-thick piece of formalin-fixed human brain tissue was incubated in OPTIClear for 6 h and it was rendered transparent even without prior delipidation in SDS. The degree of tissue transparency and decolorisation was improved with prior SDS delipidation (Fig. 1a, upper panel).

The fact that OPTIClear alone is sufficient to render a small non-delipidated piece of human brain tissue transparent supports its working principle based on compartment-selective refractive index modulation. Furthermore, thick blocks (>5 mm) of human brain tissue, which remained opaque after 3.5 months of delipidation, were rendered transparent after further incubation in OPTIClear for 6 h (Fig. 1a, lower panel). Despite OPTIClear’s high tissue-clearing efficacy, it only causes minimal structural disturbance to the tissue down to the ultrastructural level (Supplementary Figure 3).

OPTIClear is compatible with most fluorescent dyes; however, it decreases fluorescence for fluorescein, AlexaFluor488, Dylight dyes and rhodamine, requiring higher laser power for imaging compared to in PBST buffer (Supplementary Figures 4 and 5, Supplementary Table 4). We dissected the components of OPTIClear and found iohexol to be responsible for the poor fluorescence signal with xanthene dyes (Supplementary Figure 6). As expected, other tissue-clearing agents containing iodinated radiocontrasts also have a reduction in fluorescence signal. Interestingly, the lower the concentration of iohexol, the better the fluorescence signal is preserved, as demonstrated here with OPTIClear (Supplementary Figure 6b). In addition, to see whether such decrease in fluorescence would lead to increased bleaching, we found it reassuring that OPTIClear (along with some other tissue-clearing agents) did not cause increased bleaching of fluorescein and AlexaFluor568 (Supplementary Figure 6c).

### Compatibility of OPTIClear with immunofluorescent staining

We have tested the compatibility of OPTIClear in human brain immunohistochemistry using a variety of different antibodies, including GFAP (glial fibrillary acidic protein), NF (neurofilament), TH (tyrosine hydroxylase), Iba-1, ZO-1 (zonula occludens-1), HLA DQ/DR/DP (CR3/43), alpha-synuclein (αSN), tau, synapsin 1, calretinin and choline acetyltransferase (ChAT) (Supplementary Figure 7). In this study, we were not able to successfully stain for ChAT, αSN and calretinin, with or without SDS delipidation for 5 days. It is interesting to note that Iba-1 staining was not possible after 5 days of treatment with SDS.

We have also compared the clearing capability and fluorescence signal retention between OPTIClear and four other existing clearing solutions including CUBICScale, PROTOS, ScaleS and RIMS (Supplementary Figure 8). The performance of OPTIClear was similar to other solutions tested, but the tissue clearing performance was best with CUBICScale-clearing and PROTOS-clearing solutions. However, when tissues were re-imaged after 3 days of incubation in the clearing solution, OPTIClear had the best signal preservation compared with other solutions. We further show that fine structures such as dendritic spines, as well as endogenous fluorescence (YFP), immunofluorescence with AlexaFluor488 and lectin with Dylight488 were preserved in OPTIClear for >500 days at room temperature (Supplementary Figure 9); however, the signal intensity of fluorescent staining decreased and thus we recommend storage of stained tissue in PBS rather than OPTIClear.

### Tissue clearing for archival materials

Using OPTIClear and a prolonged delipidation treatment with SDS (>3 months), we performed 3D immunohistochemistry on archival tissues that have been extensively formalin-fixed for up to 50 years. We were able to achieve 300 µm deep immunostaining with good structural preservation on a piece of 2 mm-thick cingulate cortex from the Corsellis collection (with formalin fixation over 30 years). Using antibodies against GFAP, fine processes of the astrocytes forming the blood–brain barrier were well-visualised under high magnification in 3D (Fig. 1b, Supplementary Figure 10a).

We have also explored the use of FFPE tissue in tissue clearing (Supplementary Figures 10b, c). Tissue was first dewaxed with heat and xylene before rehydration in decreasing gradients of tetrahydrofuran. Staining with anti-GFAP showed fine astrocytic processes can be visualised clearly and individually traced (Fig. 1c), indicating excellent preservation of tissue structures following retrieval of FFPE tissues.

### Non-immunohistochemical staining methods for tissue clearing

**Fig. 2:**
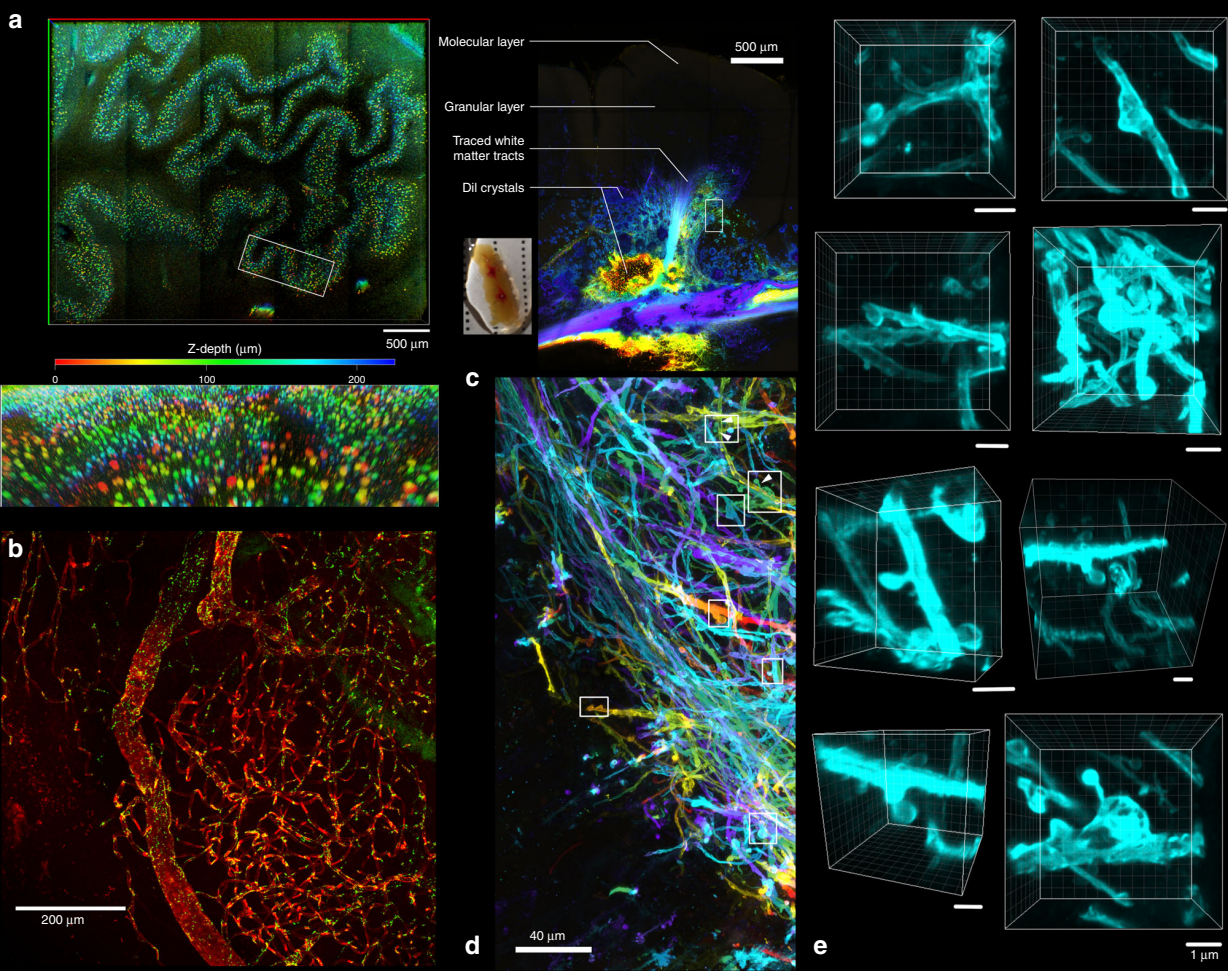
Non-immunohistochemical staining with tissue clearing on formalin-fixed brain tissues. **a** Colour depth-coded image with maximum intensity projection of a piece of 1-mm-thick medulla block stained with cresyl violet and cleared with OPTIClear (z-stack depth = 228.83 µm). Lower image showing a zoomed and tilted view of area outlined by the white box. **b** A piece of frontal cortical block immunostained for ZO-1 (green) and counterstained with DyLight 649-labelled *Lycopersicon esculentum* lectin (red). **c** DiI crystals were inserted into a cerebellar folium to trace the fibres up to the granular layer, followed by tissue clearing with OPTIClear for 6 h at 37 °C. Most of the fibres traced were mossy fibres. The inset shows the gross appearance of the sample with red crystals of DiI inserted within the OPTICleared sample. **d** Enlarged view of the white boxed region in a. with colour depth-coding (Z-depth 151.09 μm). **e** *Z*-stack images of dendritic spine-like projections visualised in areas outlined by white boxes. Scale bars, 1 μm

We screened through various traditional dyes used in routine histology and identified cresyl violet acetate as a useful pan-neuronal marker as it emits red fluorescence upon excitation with the Argon laser (488 nm). Further optimisation of the staining protocol, inspired by system-wide binding kinetics control and staining regression in traditional histology, achieved a desirable staining contrast and quality (Supplementary Methods, Supplementary Figure 11). Formalin-fixed tissue can be readily stained with cresyl violet and be rendered transparent using OPTIClear without the need for SDS delipidation. Using such a method, a piece of formalin-fixed tissue can be stained and visualised within ~5 h. Cresyl violet staining is also applicable to prolonged formalin-fixed or FFPE tissues after SDS treatment. The distribution of cresyl violet staining gives a specific subcellular staining pattern resembling that normally seen for Nissl substance, namely a perinuclear distribution with extension in the dendrites. The use of cresyl violet in tissue clearing is most useful for visualising magnocellular neurons. For example, in the inferior olivary nucleus, where a specific molecular marker for the magnocellular neurons is lacking, cresyl violet staining can effectively demonstrate their distribution in 3D space (Fig. 2a).

Fluorescently conjugated *Lycopersicon esculentum* lectin was another non-immunohistochemical marker compatible with OPTIClear. It stains blood vessels within brain tissue and could be used as a useful counterstain with immunofluorescence. In this study, a piece of tissue fixed in formalin for 45 years immunostained for zona occludens-1 (ZO-1), was counterstained with DyLight 649-labelled lectin, demonstrating the intricate blood vessel network in the brain (Fig. 2b).

### 3D neuronal tracing using lipophilic tracer

Since OPTIClear is detergent-free and denaturant-free, it is compatible with lipophilic tracers, enabling 3D visualisation of neuronal projections in rodent and human brain tissue. The tracings remained stable even after storing in OPTIClear for 2–3 weeks (Supplementary Figures 12, 13). In this study, DiI crystals were embedded in the deep white matter of a piece of cerebellar tissue for 10 days achieving a diffusion distance of 3–4 mm (Fig. 2c). Tissue was subsequently immersed in OPTIClear solution for 6 h and sparsely labelled mossy fibres in a single folium were visualised under confocal microscopy (Fig. 2d). A large number of dendritic spine-like projections in the human brain were visualised in 3D (Fig. 2e) for which computer surface rendering can be performed (Supplementary Movie 1).

### 3D neuroanatomy of the human spinal cord and cerebellum

**Fig. 3:**
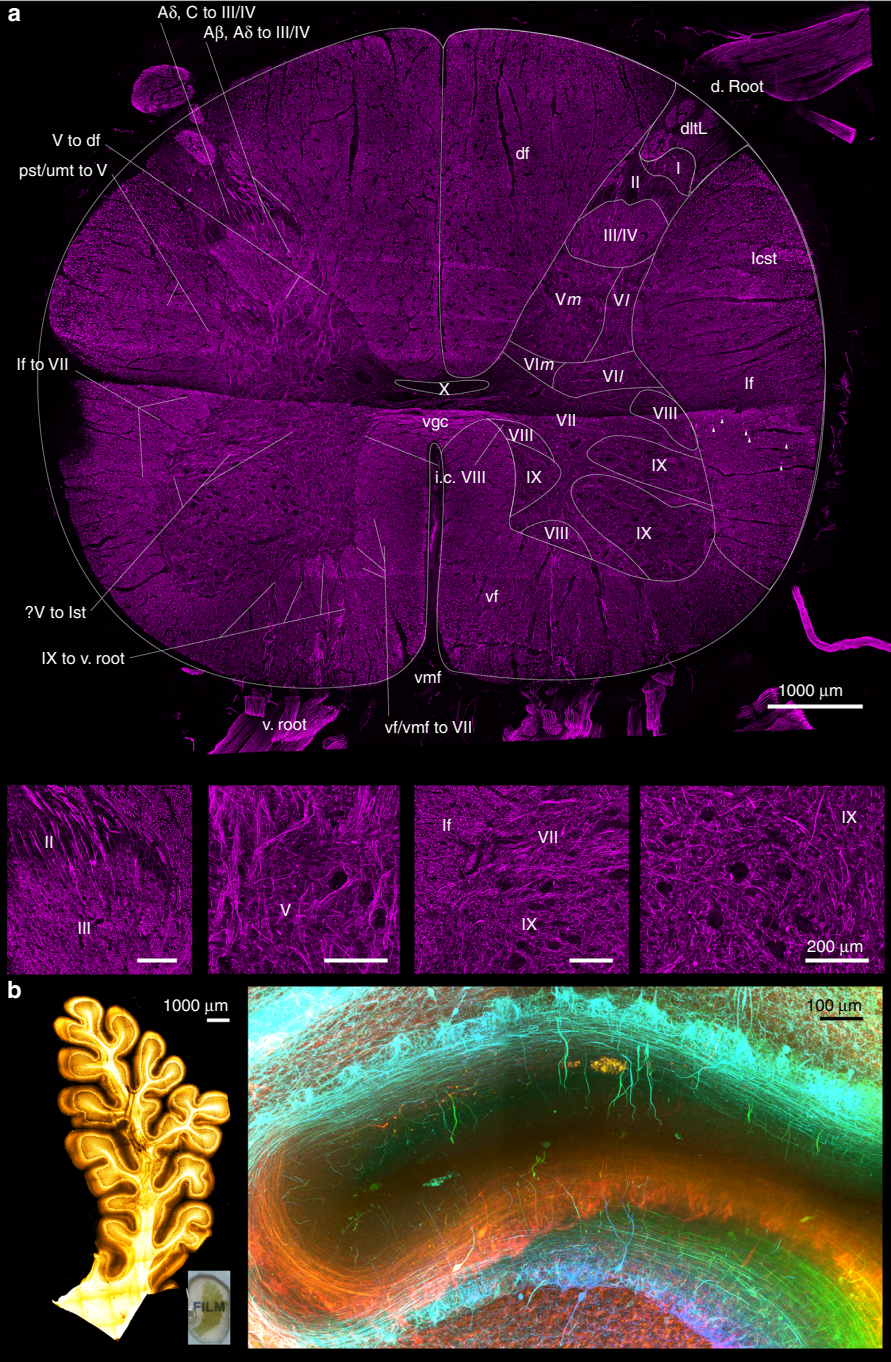
Application of 3D immunohistochemistry with next-generation histology. **a** Maximum intensity projection image of a 9.5 mm-wide, 8.1 mm-tall, 2 mm-thick spinal cord block stained for neurofilament (imaging Z-depth = 462.60 µm). Magnified view of different laminae was shown in lower images (Scale bars = 200 µm). Roman numerals from I to X: laminae of Rexed, where the suffix m and l denotes the medial and lateral divisions, respectively. Aβ, Aδ to III/IV Aβ-type and Aδ-type input fibres from dorsolateral tract to lamina III/IV, Aβ, C to III/IV Aβ-type and C-type input fibres from dorsolateral tract to lamina III and IV of Rexed, df dorsal funiculus, d. root dorsal root, i.c. VIII interconnecting fibres between bilateral laminae VIII of Rexed, IX to v. root: output motor fibres from lamina IX of Rexed to ventral root, lcst lateral corticospinal tract, lf lateral funiculus, lf to VII lateral funiculus fibres input into the lamina VII of Rexed, pst/umt to V propriospinal tracts or upper modulating tracts input into the lamina V of Rexed, v. root ventral root, ?V to lst fibres from the lamina V of Rexed on the right side coursing through the grey matter to form the lateral spinothalamic tract of the left sid, V to df output tracts from lamina V of Rexed into the dorsal funiculus, vf ventral funiculus, vf/vmf to VII input fibres from the ventral funiculus or ventromedial funiculus into the lamina VII of Rexed, vgc ventral grey commissure, vmf ventral median fissure**b**. A block of 17.6 mm-wide, 9.5 mm-tall, 1.5 mm-thick formalin-fixed cerebellar folium delipidated with SDS for 3.5 months, immunostained for neurofilament, and cleared with OPTIClear. The inset shows the gross appearance of the folium. Overview of immunostaining (right) and detailed view demonstrated by colour depth-coded image (below; Z-depth = 546.00 µm) from the white boxed area on the right.

A 2 mm-thick lumbar spinal cord section was incubated in SDS for 7 days and immunostained with antibodies for neurofilament to visualise its cytoarchitectural elements. With a total processing time of 10 days, we were able to clearly demonstrate the 10 laminae of Rexed based on their distinctive cytoarchitectural characteristics, as well as tracing inputs and outputs into each lamina (Fig. 3a). Prolonged tissue delipidation produced better tissue transparency. As an example, a 1500 µm-thick piece of cerebellar tissue was SDS treated for 3.5 months, immunostained for neurofilament, and incubated in OPTIClear solution for 3 h. Numerous thick immunopositive neuronal processes, which are occasionally continuous with some immunopositive soma, were visualised in detail (Fig. 3b).

### Detailed mapping of the brainstem catecholaminergic system

**Fig. 4:**
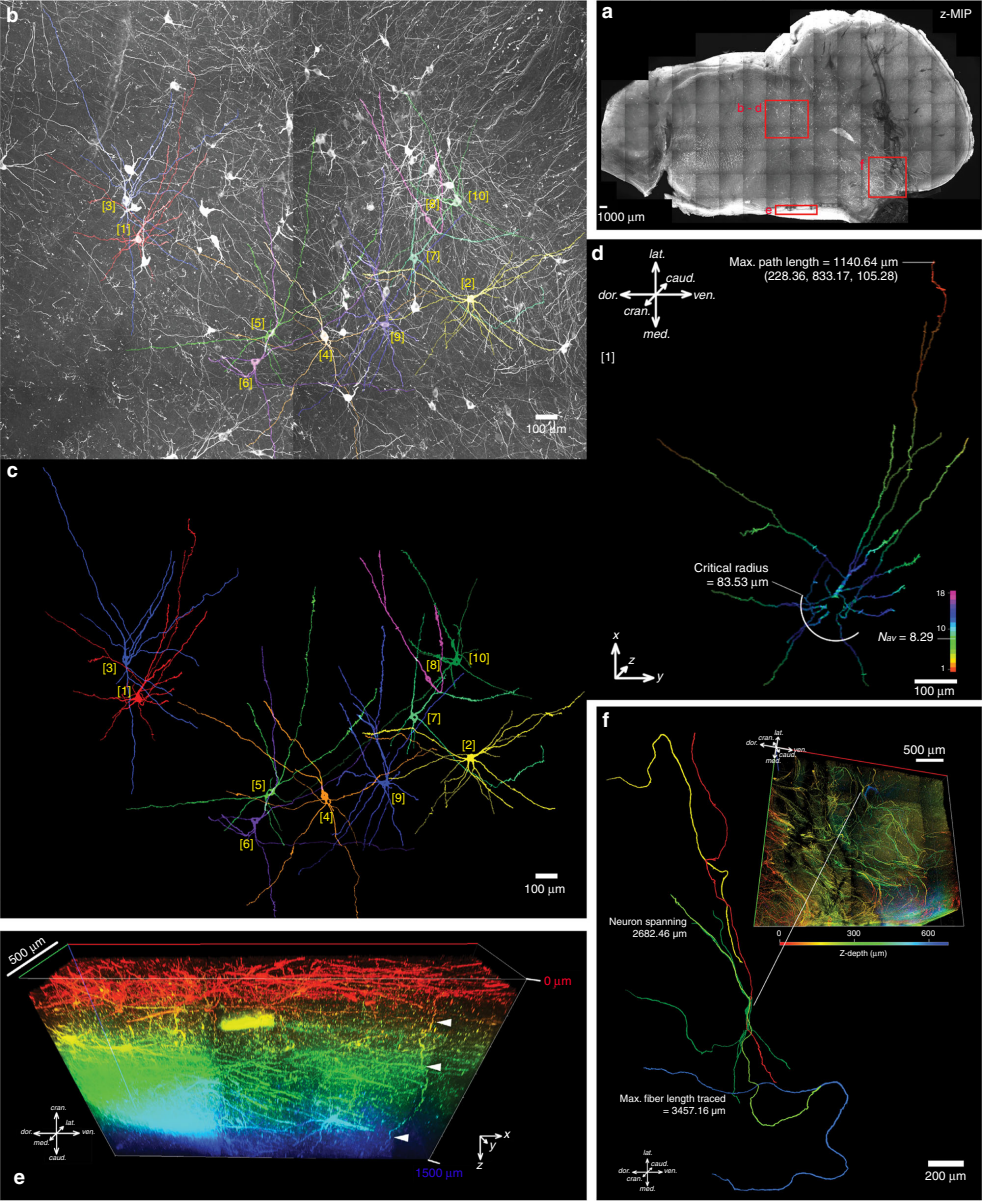
3D mapping of the brainstem catecholaminergic system. **a**. A maximum projection image of a 21.6 mm-wide, 12.2 mm-tall, 1.5 mm-thick slice of midbrain immunostained for tyrosine hydroxylase (TH) with an imaging depth of 1748.36 µm. Zoomed images showing detailed morphology of TH-immunopositive neurons. Highlighted areas are presented with analyses in subsequent subpanels. **b**–**d** Reticular formation pictured in **a**, **b** maximum projection image with 10 neurons traced and segmented, which are presented in **c**, based on which Sholl analysis has been performed and the results for neuron [1] shown in **d**. The results for other neurons is presented in Supplementary Figure 14. The cartesian coordinates (in µm) were labelled for the longest path length with the soma marked as the origin. The number of intersections was colour-coded. Nav is the average number of intersections of the entire arborisation, and is computed by dividing the area under the fitted polynomial function by the maximal radial extent of the neuronal fibres. The critical radius is radial distance from soma where the maximum number of intersections occurs. **e** The medial border of the sample in the anatomical location of caudal linear nucleus, where the antibody penetration was adequate for image throughout the thickness of the sample. This is exemplified by a fibre (highlighted by arrowheads) travelling caudocranially along the Z-direction. Z-depth colour-coded 3D rendering, scale bar on the left upper corner is 500 µm. **f** A Z-depth colour-coded 3D rendering of the substantia nigra reticulata demonstrating curliform TH-positive fibres as they are being separated by the descending tracts from the cortex. Some of these has been traced demonstrating fibre lengths and neuronal span up to 3457 µm and 2682 µm, respectively

3D immunohistochemical staining using anti-tyrosine hydroxylase (TH) antibody was used for the mapping of catecholaminergic cell groups in the human midbrain block. In a 2 mm-thick hemi-midbrain slice from a healthy donor with SDS delipidation of 3.5 months, our antibody staining achieved around 250 μm penetration from both surfaces, which enabled the visualisation of many cell groups at once as well as continuous, traceable TH-positive fibres. Using this method, cellular morphology and fibre projections were clearly visualised. We were able to stain for TH-positive neurons located within the substantia nigra pars compacta and its associated paranigral nucleus, ventral tegmental area, parabrachial pigmented nucleus, mesencephalic reticular formation, caudal/rostral linear nucleus and dorsal raphe nucleus, with their detailed morphologies including third-order axonal branches visualised all at once (Fig. 4, Supplementary Figure 14).

### Visualisation and quantification of basal forebrain neurons

**Fig. 5:**
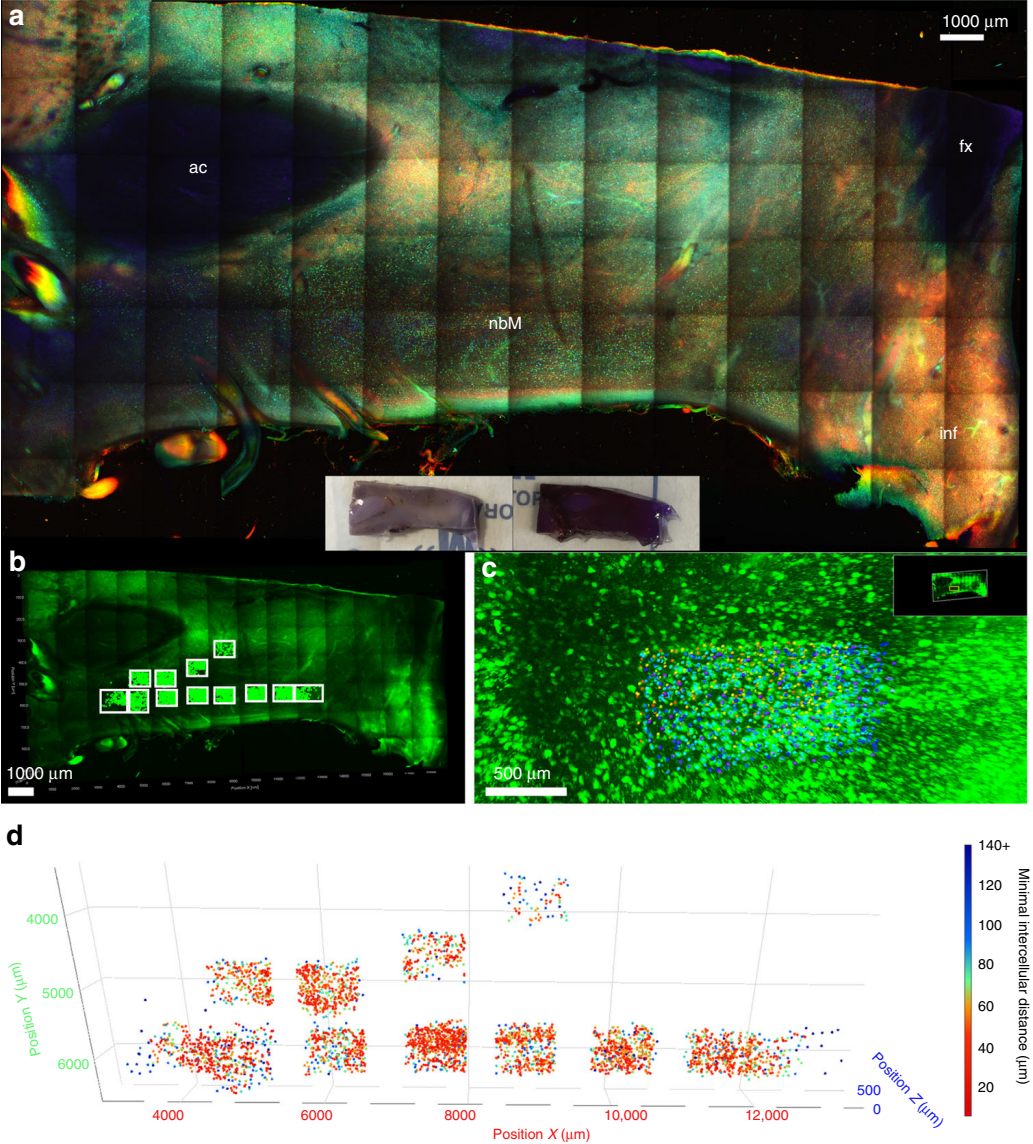
3D visualisation and quantification of basal forebrain magnocellular neurons. **a** Three-dimensional cresyl violet staining for the visualisation of the nucleus basalis of Meynert (nbM) on a 10.8 mm-wide, 5.5 mm-tall, 2.5 mm-thick basal forebrain block (imaging Z-depth = 1120.00 µm). Tissue block was delipidated with SDS for 3 days and stained with cresyl violet for 3 days (gross appearance shown in inset). Image on bottom left showed magnified view of selected areas from top image. Z-positions of the quantified and annotated neurons were colour-coded according to the reference bars, where the numerical values indicate the distances from the imaging objective for each end of the spectrum (bottom right). **b–d** Quantification of magnocellular neurons in region of interests highlighted in **b** (white boxes), where the coordinates of each neuron were marked and annotated (**c**). **d** 4D scatter plot of all 3528 neurons, the distance of each neuron to its nearest neighbour was calculated and represented with colour coding. More detailed statistics were provided in Supplementary Figure 15. ac anterior commissure, fx fornix, inf infundibulum, nbM nucleus basalis of Meynert

We performed 3D histological staining using cresyl violet on a 2.5 mm-thick piece of basal forebrain tissue containing the nucleus basalis of Meynert (nbM) in a healthy control. With a total processing time of 5 days (3 days of SDS delipidation, 1 day of staining and 1 day of incubation in OPTIClear), we were able to image down to a total depth of 1000 µm (500 µm on each side). The staining highlighted regions densely populated by neurons, leaving white matter or myelin-rich regions dark. Using this technique, we were able to visualise how magnocellular neurons of the nbM were arranged spatially in relation to other anatomical structures (Fig. 5a). With automated annotation, individual neurons were assigned a spatial coordinate and the inter-cellular distance calculated (Fig. 5b, Supplementary Figure 15).

## Discussion

**Fig. 6:**
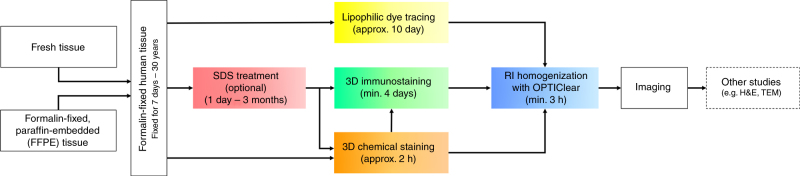
Flowchart summarising the next-generation histology protocol developed in this paper. The protocol outlined here enables both fresh and archival formalin-fixed paraffin-embedded (FFPE) post-mortem tissues to be processed for tissue clearing. Formalin-fixed tissues can then be stained using immunohistochemical or non-immunohistochemical techniques such as lipophilic dye tracing with DiI or 3D chemical staining with cresyl violet. An optional SDS delipidation step is recommended before 3D immunostaining or chemical staining. Subsequently, tissues are rendered optically transparent with refractive index homogenization using OPTIClear before imaging or proceeding to other studies such as electron microscopy. H&E, haematoxylin & eosin; RI, refractive index; SDS, sodium dodecyl sulphate; TEM, transmission electron microscopy

Despite the multitude of brain tissue-clearing techniques currently available, none were originally developed or optimised on human pathological samples. Compared to rodent brain tissue, human tissue has a considerably longer processing time^5,16^. Moreover, due to the prolonged formalin fixation in most archival materials, the quality of immunostaining is often poor due to antigen masking by excessive formaldehyde protein crosslinks^17^. Previously, we developed a more efficient tissue-clearing protocol which improved immunostaining in human brain samples, and it greatly reduced the complexity and speed of the delipidation-based clearing techniques by eliminating the acrylamide-embedding step^8^. In this study, we have further optimised the technique and developed a selection of different strategies for human tissue-clearing research (Fig. 6, Supplementary Movie 1). On the basis of the concept of compartment-selective refractive index adjustment, a refractive index homogenising agent, OPTIClear, has been developed. This solution is a detergent-free and denaturant-free formula that is thought to alter only optical properties of the tissue with minimal structural and molecular disturbances. This has enabled an efficient tissue-clearing protocol and it is compatible with different molecular labelling methods. These include its compatibility with lipophilic tracing, and rapid visualisation of cytoarchitecture using non-immunohistochemical staining methods such as cresyl violet and lectin staining. Interestingly, iodinated radiocontrasts would decrease the fluorescence of many xanthene dyes. Since iodinated radiocontrasts are the principal components of many tissue-clearing formulae, the search for alternatives or formulae with lower concentrations of iodinated radiocontrasts may lead to further development in tissue clearing.

Immunostaining with tissue clearing using human tissues remains challenging. Although we demonstrated good staining with antibodies against a variety of antigens, staining remained difficult for some (including calretinin and ChAT). Also, antigens such as Iba-1 may be masked following treatment with SDS. This prompted further work in the optimisation of immunostaining in human brain tissues for tissue clearing. We discovered that, although the clearing performance of OPTIClear, CUBICScale and PROTOS were similar, OPTIClear had a better preservation of fluorescent signal.

Using the optimised protocol, we can now perform tissue clearing on prolonged formalin-fixed and paraffin-embedded tissues. One practical use is that tissues can then be processed for 3D analysis after routine diagnostic workup (with standard histological and immunohistochemical staining on tissue sections) is performed. This provides a huge potential for accessing and using diagnostic archives for research purposes, particularly for rare neurological disorders. It is important to note, however, that although staining was possible, the immunostaining quality, antibody penetration depth and structural preservation were not as good as standard formalin-fixed tissues when treated with the same tissue-clearing and immunostaining procedures.

Unlike in rodents (where the maximal size of tissue is the whole brain), human brain tissue from different regions can vary in size and tissue composition such that the optical properties and ease of SDS delipidation may vary. Although duration of formalin fixation can be standardised, pre-mortem agonal state of the patients, post-mortem changes such as pH variation and oxygenation of tissue and pathology within the brain (e.g., sclerosis or necrosis) will mean tissue quality varies considerably from one brain to another. As a result, we investigated clearing thickness and imaging depth of incubation with OPTIClear solution with varying duration of SDS treatment. First, we were able to achieve a clearing thickness of 1 mm for samples fixed for 1 week (Fig. 1a, upper panel) without delipidation. Second, an imaging depth of 150 µm was achieved with lipophilic tracer (limited by working distance, Fig. 2d) for samples fixed for 1 week without delipidation. Third, an imaging depth of 500 µm was achieved with 3 days of delipidation with cresyl violet staining for samples fixed for 3 weeks (Fig. 5). Finally, an imaging depth of 1500 µm was achieved with 3.5 months of delipidation for samples fixed for 1 week with immunofluorescence (Fig. 4). In a recent study using a modified CLARITY protocol (hydrogel embedding with 2% acrylamide/0.05% bisacrylamide), human brain samples were cleared and imaged down to 5 mm in depth with both active (electrophoretic) and passive SDS clearing for ~10–14 months. The tissue remained structurally intact even after prolonged SDS clearing. Similar to our present study, tissue block was optically opaque and only became visibly transparent after refractive index homogenisation with 47% TDE^18^. These studies demonstrated flexibility in the timing of SDS clearing, while maintaining structural integrity for morphometric analyses (Figs. 4 and 5) regardless of the fixation duration, SDS treatment duration and the brain region sampled. Hence, this exemplifies an important concept in the development of next-generation histology, that refractive index homogenisation (in this case with OPTIClear) is the most important step in optical clearing. While SDS treatment (used in CLARITY) can be used as an adjunct, it is not a necessary step for tissue clearing with OPTIClear, instead we hypothesise that it has a crucial role to ensure adequate tissue permeabilization and antigen retrieval for immunohistochemical staining. Furthermore, on a practical note, we observed that the depth of antibody penetration depended more on the density of the antigens and the amount of antibody added, rather than on the incubation time. Further investigations on the factors governing antibody penetration depth and staining quality are thus necessary. In general, given sufficient amount of antibody, we observed that a permeabilisation step with PBST would allow antibody penetration of at least 200 μm.

In routine pathology practice, the use of structural stains such as haematoxylin and eosin (H&E) or Luxol fast blue with cresyl violet (LFB/CV) is important for tissue orientation by identification of key anatomical landmarks. However, so far only immunofluorescence staining has been performed on cleared human brain tissues^4,5^. We have developed a protocol for the use of cresyl violet staining on tissue blocks which can subsequently be cleared using OPTIClear. This stained neuronal somata and enabled the visualisation and quantification of neurons in the 3D space. Similar to histological staining on tissue sections, cresyl violet staining with tissue clearing is simple and rapid—a small piece of formalin-fixed tissue block could be stained and readily visualised within 5–6 h using this method. In this study, we used cresyl violet staining on a block of basal forebrain tissue to visualise the distribution of cholinergic magnocellular neurons in the nbM. Normally, cholinergic neurons are identified using antibodies against ChAT, an enzyme required for acetylcholine synthesis^19^. However, immunostaining with anti-ChAT antibodies has proven challenging on human brain tissues and staining intensity is greatly affected by pre-mortem and post-mortem variables^20^. Since over 90–95% of magnocellular neurons within the nbM are ChAT immunopositive^21^, the use of Nissl staining with cresyl violet for the identification of the basal forebrain cholinergic population is justified. We have demonstrated that a 1 mm block of human basal forebrain tissue, containing the nbM, can be delipidated, stained and visualised within 5 days. This method of staining is particularly useful on labelling magnocellular neurons, which are also found in brainstem nuclei such as the inferior olivary nucleus. When combined with colour depth-coding, stained neurons could be individually identified and full quantification of an area of interest could be performed. The application of this technique could potentially replace the current practice of stereology for the quantification of neurons in a discrete nucleus or region of interest.

For immunofluorescence in tissue clearing, one tends to use nuclear stain, e.g., DAPI, as a counterstain for spatial orientation under the confocal microscopes^3,5^. Here apart from cresyl violet staining, we demonstrated staining with fluorescent-labelled lectin can be used as a counterstain in immunofluorescence studies to visualise blood vessels in brain tissue blocks. This acts as a structural landmark which is another method to aid orientation of tissue in the 3D space. It is also suitable in studies involving the blood–brain barrier, such as the investigation of endothelial tight junction as demonstrated in the present study using immunofluorescence staining to ZO-1.

Transgenic animals with fluorescent protein-labelled neurons, e.g., Thy1-YFP mice, can be used to trace axonal and dendritic processes of an individual neuron in its entirety^22^. When combined with tissue-clearing technology, this can be very powerful in the study of anatomical and structural connections of the brain^3^. However, in humans, neuronal tracing can only be performed post-mortem and this has been demonstrated using Golgi stain or the carbocyanine dye, DiI^23^. Since DiI is a lipophilic tracer, it is not compatible with existing tissue clearing techniques which involve the use of organic solvent or delipidation. With the use of OPTIClear, the lipid membranes are well-preserved, such that neuronal processes of a block of cerebellar tissue could be successfully traced with DiI. For the first time, human dendritic spine-like projections could be visualised in 3D and the morphology is comparable to previous studies on human hippocampal sections^23,24^, a goal that has not been achieved by other tissue-clearing techniques or traditional histology.

Use of the refined tissue clearing technique and 3D histology can complement existing human anatomical atlases. Neuropil and neuronal soma have long been used for the definition of neuroanatomical areas or regions since the time of Ramόn y Cajal^25^. Using immunostaining for neurofilament with tissue clearing on a thick tissue block, a detailed perspective on the cytoarchitectural elements that define various anatomical regions can be visualised. Since the size of the neurofilament in immunohistochemistry correlates with the actual axo-dendritic diameter, it provides a convenient approach to the estimation of type of neuronal fibres^26^. Hence, we were able to create a fully annotated cross-section of a piece of human spinal cord in this study with the 10 laminae of Rexed clearly identified. In the human caudal midbrain, we have demonstrated that complete mapping of a neurotransmitter system is possible. TH-positive cells and their projections can be labelled and traced over great distances. Normally, the demonstration of cellular morphology and tracing of fibre projections is often not possible with conventional histology due to the substantial tissue deformation that leads to difficulties in registering individual fibres across sections. Also, array tomography with in silico reconstruction is unsuitable for such studies due to the limits in sectioning surface area for epoxy resin-embedded samples^27^. In this study, immunostaining of TH in the human midbrain block was comparable to a previously published atlas of catecholaminergic neurons in human^28^. However, the somal branching pattern appeared more extensive (Fig. 4) and fine TH-positive fibres, over a Z-depth of 1500 μm, or up to 3000 μm in length, can be readily traced. (Fig. 4, Supplementary Figure 14). These groups of fibres could have been missed in earlier studies, as the TH-positive fibres were characteristically sparse, particularly in thin histological sections. This demonstrates the advantage of the current technique over standard histological studies and highlights the importance of revisiting older structural anatomical studies.

Despite potential applications of tissue clearing in human tissues identified in the current study, there are still several issues that remain to be resolved. Autofluorescence in human tissues and prolonged fixation of post-mortem material are difficult issues to tackle, and so far the use of conventional reagents that reduces autofluorescence in tissue sections have failed to work as effectively in thick tissues^29,30^. Although prolonged incubation in SDS helps, speeding up tissue decolorisation can enhance flexibility of the process. Effective immunohistochemistry in cleared thick tissue blocks remains a challenge^9^, particularly in human brain tissues which have been extensively fixed. Antibody penetration depth can be inconsistent and although several methods have been proposed to overcome this issue in animals, including the use of electric fields^31^ and system-wide binding kinetics controlling agents^29^, we have found that they are either inapplicable to human tissues, incompatible with double immunofluorescence and indirect immunohistochemistry, or that they lead to significant non-specific staining. Regardless of which approach is taken, the large amount of antibodies needed for large scale neuropathological studies can be a limitation and therefore the development of alternatives such as chemical probes and aptamers needs further attention. Finally, the immunostaining quality of the same antibodies in conventional slices and delipidated tissues can occasionally differ significantly, thus immunostaining and antigen retrieval conditions need optimisation for each antibody individually.

In summary, we have developed a selection of protocols for the study of human brain tissue in 3D, which are flexible and versatile for use by histopathologists to address different research questions concerning microstructural anatomy in health and disease. These strategies are simple to execute and will hopefully provide the basis for further technique development.

## Methods

### Next-generation histology pipeline

The next-generation histology pipeline consists of appropriate tissue retrieval and preservation methods, an aqueous-based tissue-clearing method with optional tissue delipidation, 3D immunofluorescence, 3D histochemical staining, lipophilic dye tracing and imaging, after which the sample can be restored by washing in PBST at 37 °C for another round of next-generation histology processing. A detailed, step-by-step protocol has been uploaded to Protocol Exchange. Example tissue processing schemes have been provided in Supplementary Table 3 for the figures presented in this study.

### Collection and preparation of human brain tissue

The tissue used in this study was provided by the Parkinson’s UK Tissue Bank at Imperial College London and by the Corsellis archival collection. Fresh brain tissue blocks of approximately 1 cm^3^ in size were collected from the Parkinson’s UK tissue bank and were fixed in 10% buffered formalin for one week before proceeding. Blocks of routine diagnostic tissue, taken from whole or hemisected brains fixed in formalin for 3–4 weeks, were also used. Wet tissue taken from Corsellis collection cases had been fixed in formalin for at least 30 years.

Archival FFPE brain tissue was also used in this study. Excess paraffin wax was carefully trimmed away using a scalpel followed by incubation in a 60 °C oven until all the wax was molten. The tissue was then incubated in xylene at 60 °C overnight and rehydrated through a graded series of tetrahydrofuran solutions (100, 80, 50, 20%) for 1 h each at room temperature. The retrieved tissue was then treated in the same way as standard formalin-fixed tissue for processing as detailed below.

### Immunofluorescence staining

Immunostaining of processed tissue blocks followed our previously published FASTClear protocol^8^. First, formalin-fixed tissues were dissected into smaller blocks (~5 mm in thickness) and were immersed in 4% SDS (dissolved in 0.2 M sodium borate, pH 8.5) at 55 °C for delipidation. The duration of SDS treatment varied and was dependent on the extent of tissue discolouration, the desired transparency, the size of the tissue block and the antigens to be detected. As a rule of thumb, (i) the longer the tissue fixed in formalin, the longer the SDS treatment recommended; (ii) White matter regions require longer SDS treatment (at least 10 days is recommended) than grey matter; (iii) for the specific antibody used, the harsher the antigen retrieval condition required in standard immunohistochemistry on tissue sections, the longer the SDS treatment recommended for intact tissue blocks; and (iv) the longer the SDS treatment, the less brownish discoloration, autofluorescence and non-specific binding of antibodies to lipofuscin. For example, for the immunostaining of neurofilament on routine diagnostic tissues, 5–7 days of SDS delipidation is normally sufficient. For prolonged formalin-fixed materials, a delipidation time of ~3 months may be required. Note that after SDS delipidation, complete transparency of the tissue block is not required.

Next, the tissue was washed in phosphate-buffered saline (PBS) with Triton X-100 (PBST; 0.1% Triton X-100 (vol/vol) and 0.01% sodium azide (wt/vol)) at 37 °C overnight. The tissue was then immunostained with primary antibody in PBST at 37 °C for at least 2 days, with daily supplementation at a dilution of 1:100. The amount of antibody used, as well as the primary antibody incubation period can be extended if necessary. 1% bovine serum albumin or 6% normal donkey serum can also be added if deemed necessary to reduce non-specific staining. For most antibodies, a starting concentration of 1:100 could be used. For densely expressed antigens (e.g., glial fibrillary acidic protein, GFAP), we recommend a starting concentration of 1:500 with daily supplementation at 1:100 for 1 week. All antibodies used in this study are listed in Table 1 below.

**Table 1:** Antibodies used in this study

Antibody	Host	Clonality	Immunogen	Company	Catalogue number
Alpha-synuclein (Clone 42)	Mouse	Monoclonal (IgG1)	Rat Synuclein-1 aa. 15-123	Becton Dickson (BD) Transduction Laboratories	610787
Calretinin	Goat	Polyclonal	Rat calretinin	Millipore	AB1550
Choline acetyltransferase (ChAT)	Goat	Polyclonal	Human placental ChAT	Millipore	AB144P
Glial fibrillary acidic protein (GFAP)	Rabbit	Polyclonal	GFAP isolated from bovine spinal cord	Agilent Dako	Z0334
Glial fibrillary acidic protein (GFAP)	Rabbit	Polyclonal	GFAP from human brain	Sigma	G9269-0.2 ML
Ionised calcium-binding adapter molecule 1 (Iba-1)	Rabbit	Polyclonal	Synthetic peptide corresponding to C-terminus of Iba1	Wako	019-19741
Neurofilament (NF, Clone 2F11)	Mouse	Monoclonal (IgG1, kappa)	Neurofilament isolated from normal adult human brain	Agilent Dako	M0762
Synapsin I	Rabbit	Polyclonal	Native protein purified from bovine brain	Novus biologicals	NB300-104
Tau (AT8; Phospho-PHF-tau pSer202 + Thr205)	Mouse	Monoclonal (IgG1)	Partially purified human PHF-Tau	ThermoFisher Scientific	MN1020
Tyrosine hydroxylase (TH)	Rabbit	Polyclonal	Denatured tyrosine hydroxylase from rat pheochromocytoma	Millipore	AB152
Zonula occludens-1 (ZO-1)	Rabbit	Polyclonal	Synthetic peptide derived from the N-terminal region of human, dog, mouse and rat ZO-1	ThermoFisher Scientific	40-2300

The tissue was then washed in PBST at 37 °C three times over the next 24 h and incubated with secondary antibody in the same procedure as per primary antibody incubation described above. Due to the yellow–brownish discoloration of human brain tissues, fluorophores with absorbance emission spectra as bathochromatically shifted (near-infrared spectrum) as possible are recommended (e.g., Alexa Fluor 647-conjugated antibodies). A nuclear counterstain (e.g., 4’,6-diamidino-2-phenylindole, DAPI at 1 μg/ml) or fluorophore-conjugated lectin stain (1:100; DyLight 649-labelled *Lycopersicon esculentum* lectin; Catalog Number: DL-1178; Vector Labs, UK) for blood vessels can also be added in this step. After immunostaining, the sample was washed in PBST at 37 °C three times over the next 24 h before proceeding to refractive index homogenisation and imaging.

### Non-immunohistochemical staining with cresyl violet

Cresyl violet staining was performed with either delipidated or untreated formalin-fixed tissue. The tissue was incubated in 0.1% cresyl violet acetate with 3.6% SDS in borate buffer at 37 °C for at least 2 h, followed by immersion of the tissue in 0.25% acetic acid in 100% methanol at room temperature until grey–white matter differential staining was no longer present (Supplementary Figure 16). This procedure should take no longer than 30 min as it may cause precipitation of tissue proteins and subsequent difficulties in immunohistochemistry.

### Lipophilic tracer labelling with DiI

Lipophilic tracing was performed by inserting crystals of 1,1’-Dioctadecyl-3,3,3’,3’-tetramethylindocarbocyanine perchlorate (DiI) (Catalogue number: D282; ThermoFisher Scientific, UK) into the white matter of a piece of formalin-fixed brain tissue (without prior SDS delipidation). A small incision was made in the tissue to facilitate insertion of the crystals. Afterwards the tissue was immersed in PBS to keep the tissue hydrated, taking care to not dislodge the crystals and incubated at 37 °C before proceeding to refractive index homogenisation. The duration of incubation depends on the specific nature of the study. In the current study, a 3–4 mm diffusion distance was achieved with 10 days of DiI embedding in a 5-year-fixed brain.

### Refractive index homogenisation with OPTIClear

We performed chemical screening using whole-brain lipid extract liposomes and boiled egg whites as models for tissue hydrophobic and proteinaceous compartments. This led us to identify three key chemicals which can suitably adjust refractive indices of individual compartments of brain tissues. The cocktail of these chemicals is termed OPTIClear. It is an aqueous solution consisting of 20% (wt/vol) N-methylglucamine, 32% (wt/vol) iohexol and 20.48% (vol/vol) 2,2’-thiodiethanol (TDE), with a pH between 7 to 8 adjusted with hydrochloric acid.

Immunostained or non-immunohistochemically stained tissues were incubated in the OPTIClear solution for 6 h or more at 37 °C, when maximal tissue transparency is usually achieved. A volume of OPTIClear three times that of the tissue volume is usually sufficient. Gentle agitation can facilitate penetration of the reagents into the tissue. The OPTIClear solution used for refractive index homogenisation should be used as the mounting medium for subsequent microscopy. The sample can be stored in OPTIClear solution indefinitely at room temperature. For prolonged storage, the addition of 0.01% sodium azide is recommended to avoid microbial growth. One can repeat the immunostaining after restoring the sample by washing the cleared sample in 1× PBST overnight at room temperature.

### Confocal microscopy and image acquisition

Imaging of stained and refractive index-homogenised tissue was performed using a Leica SP5 Confocal Microscope (Leica, Germany) and a Carl Zeiss LSM 780 Confocal Microscope (Carl Zeiss, Oberkochen, Germany) at the Facility for Imaging by Light Microscopy (FILM) facility in Hammersmith Hospital, Imperial College London. For images acquired on the Leica SP5 microscope, a ×10 objective (Plan-Apochromat CS; numerical aperture, 0.40; working distance, 2.2 mm), ×20 objective (Plan-Apochromat CS; numerical aperture, 0.70; working distance, 0.59 mm), ×40 objective (Plan-Apochromat; numerical aperture, 0.85; working distance, 0.21 mm) and ×63 objective (Plan-Apochromat CS; oil immersion; numerical aperture, 1.40; working distance, 0.14 mm) were used. For images acquired on the Carl Zeiss LSM780 microscope, a ×10 objective (Plan-Apochromat Ph1 M27; numerical aperture, 0.45; working distance, 2.0 mm), ×20 objective (Plan-Apochromat Ph2 M27; numerical aperture, 0.45; working distance, 0.46 mm), ×40 objective (Oil immersion; DIC M27; numerical aperture, 1.40; working distance, 0.13 mm) and ×63 objective (Plan-Apochromat, oil immersion; DIC M27; numerical aperture, 1.40; working distance, 0.19 mm) were used. The laser excitation wavelengths used were 405 nm, 488 nm, 543 nm and 594 nm. For tissue stained with cresyl violet, argon laser excitation at 488 nm was used. Detection spectra range of ~570–700 nm was used to obtain good contrast with minimal detection of autofluorescence signal.

### Data analysis

Image visualisation was performed using Fiji (Image J, NIH), Zen Black (Carl Zeiss, Germany) and Zen Blue (Carl Zeiss, Germany) software; Maximum intensity projections and z-depth colour coding were performed using Fiji and Zen Black software; 3D renderings were performed using Zen Blue and Imaris (Bitplane, Belfast, UK) software; Stitching was performed using Fiji, Zen Black and Adobe After Effects (Adobe, UK). Manual neuron tracing and Sholl analysis was performed using simple neurite tracer in Fiji, while automated neuron counting was performed using the “spots” tool in Imaris.

### Ethical considerations

The work was conducted under ethical approval held by the Parkinson’s UK Tissue Bank at Imperial College London (Registered charity in England and Wales (258197) and in Scotland (SC037554); MREC approval reference number: 07/MRE09/72). Use of tissue from the Corsellis collection was under MREC approval (reference number: 14/SC/0098). Informed consent was obtained prospectively for the use of all post-mortem brain tissues and brain samples were obtained and prepared in accordance to the Wales Research Ethics Committee approved protocols.

### Data availability

The authors declare that the data supporting the findings of this study are available within the paper and the Supplementary Information file.

## Electronic supplementary material

Supplementary Information

Descriptions of Additional Supplementary Files

Supplementary Movie 1
