# Author Correction: Next generation histology methods for three-dimensional imaging of fresh and archival human brain tissues

**DOI:** 10.1038/s41467-018-05089-5

**Published:** 2018-07-10

**Authors:** Hei Ming Lai, Alan King Lun Liu, Harry Ho Man Ng, Marc H. Goldfinger, Tsz Wing Chau, John DeFelice, Bension S. Tilley, Wai Man Wong, Wutian Wu, Steve M. Gentleman

**Affiliations:** 1 School of Biomedical Sciences, Li Ka Shing Faculty of Medicine, The University of Hong Kong, Hong Kong 852, Hong Kong; 2 Neuropathology Unit, Division of Brain Sciences, Imperial College London, Burlington Danes Building, Hammersmith Campus, Du Cane Road, London, W12 0NN UK; 3 GMH Institute of CNS Regeneration, Jinan University, 601 Huangpu Avenue West, Guangzhou 510632, China; 4 Re-Stem Biotechnology Co., Ltd, 2588 Wuzhong Road, Suzhou 215310, China

Correction to: *Nature Communications* **9**:1066 10.1038/s41467-018-03359-w; published online: 14 March 2018

In the original version of this Article, the concentration of boric acid buffer for the SDS clearing solution was given incorrectly as ‘1 M sodium borate’ and should have read ‘0.2 M boric acid’. Also, the composition of PBST incorrectly read ‘1% Triton X-100 (vol/vol) and 0.1% sodium azide (wt/vol)’ and should have read ‘0.1% Triton X-100 (vol/vol) and 0.01% sodium azide (wt/vol)’. Further, the pH of the OPTIClear solution was not stated, and should have read ‘with a pH between 7 to 8 adjusted with hydrochloric acid’. These errors have been corrected in both the PDF and HTML versions of the Article.

