# Supplementary material for: Next generation histology methods for three-dimensional imaging of fresh and archival human brain tissues
Source: Nat Commun. 2018 Mar 14;9:1066. doi: 10.1038/s41467-018-03359-w (PMC5852003; doi:10.1038/s41467-018-03359-w)
Supplement: Supplementary file 1 — Supplementary Information [file 41467_2018_3359_MOESM1_ESM.pdf]

Supplementary Information

Next generation histology methods for three-dimensional imaging of fresh and archival human brain tissues

Lai, Liu and Ng et al.

Contents

1. Supplementary Figures
2. Supplementary Tables
3. Supplementary Methods

Supplementary Figures

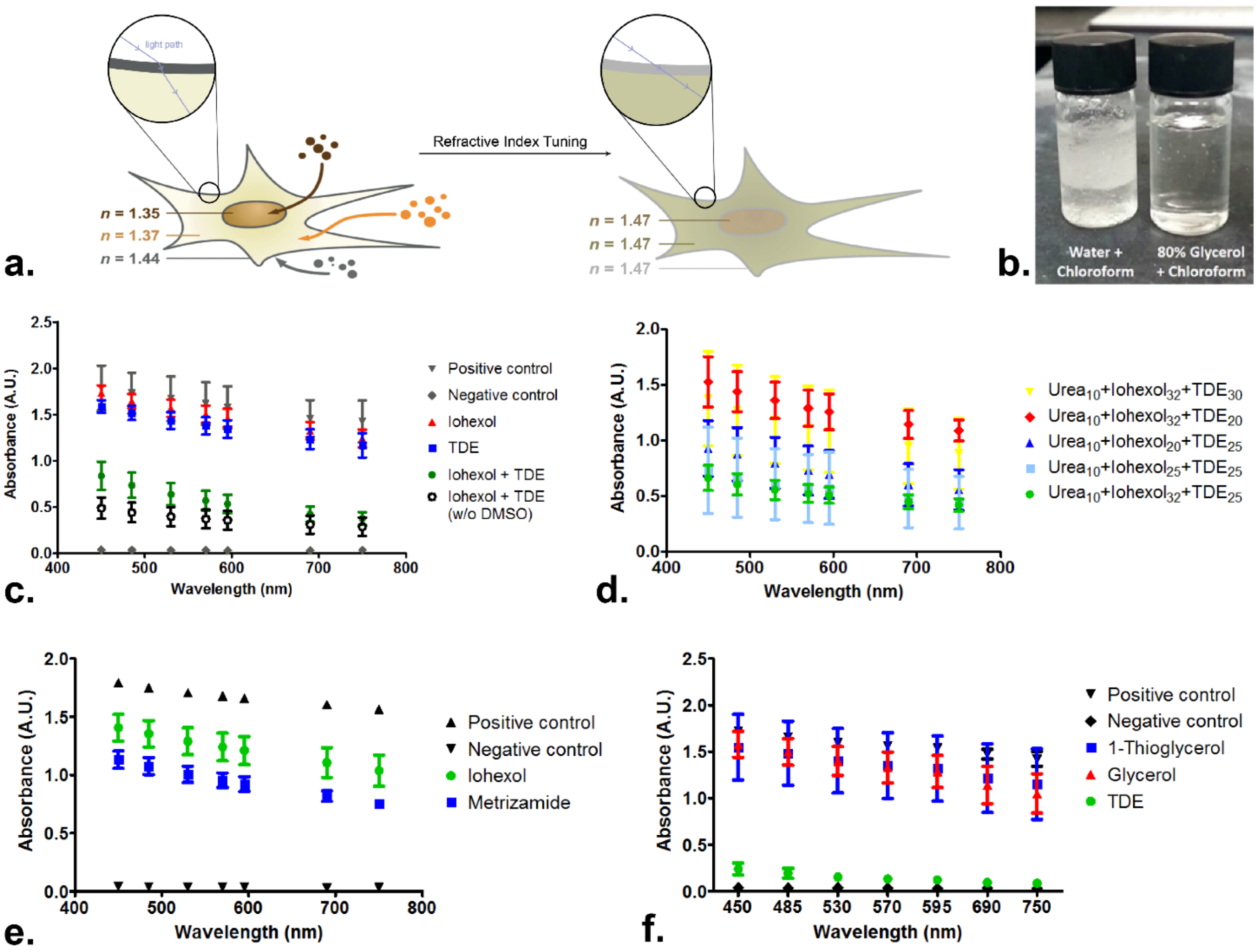

Supplementary Figure 1. Development of OPTIClear. a. Proposed tissue clearing approach by compartment-selective refractive index (RI) adjustment. The colored spheres represent reagents that would selectively dissolve in various compartments to modulate their optical properties. **b.** Simulating the effect of compartment selective RI adjustment. In the left vial, two immiscible liquids (water and chloroform) were rigorously shaken to form an emulsion, which was opaque as their RIs were poorly matched. In the right vial, 80% glycerol in water was shaken with chloroform, the resulting emulsion, despite being physically inhomogeneous, appeared transparent as the RIs of two immiscible media approximated to each other. **c – f.** Absorbance of brain homogenates after incubating in various tissue clearing formulae, PBS was used in positive controls while brain homogenate was omitted and substituted by PBS. Each entity has three samples with three repeated measurements, and the average values were plotted. Error bars are standard deviations of 3 experimental replicates. **c.** A synergistic effect was demonstrated when iohexol (at 32% w/v) and TDE (20% w/v) were used together. All formulae contained 10% urea and 25% DMSO to facilitate homogenization, except for "iohexol + TDE (w/o DMSO)" where DMSO has been omitted and surprisingly demonstrated a further reduced absorbance. **d.** Establishing the optimal concentrations of iohexol and TDE as 32% w/v and 25%, respectively. **e.** Substituting iohexol with another iodinated radiocontrast metrizamide showed that metrizamide used at the same concentration

may be superior to iohexol, but this was not the case when applied to tissue slices (data not shown). The experiment was performed with the addition of 10% urea and 25% TDE to all formulae. **f.** TDE outperformed 1-thioglycerol and glycerol when used at the same concentration. The experiment was performed with the addition of 10% urea and 32% iohexol to all formulae.

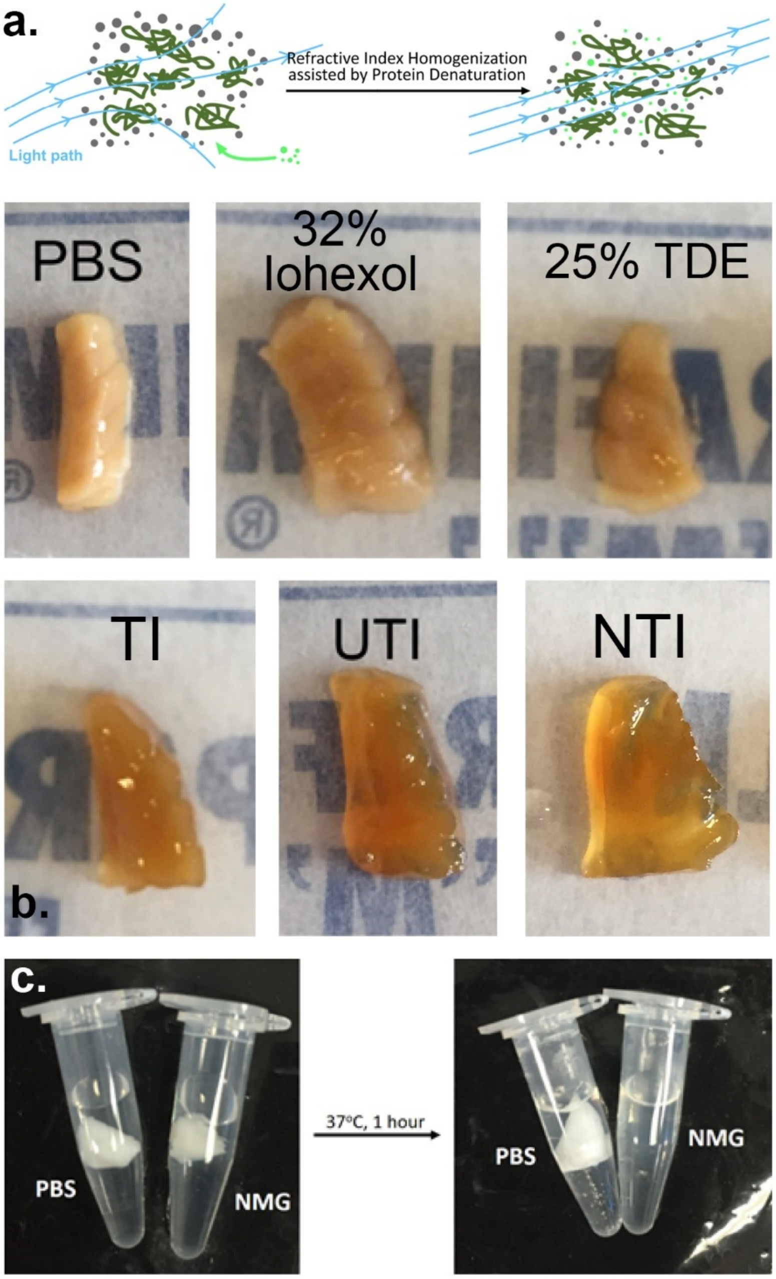

Supplementary Figure 2. Identification of *N*-methylglucamine as a suitable homogenisation facilitator. a. The addition of a homogenisation facilitator (dark green dots), such as a denaturant, may allow better infiltration of refractive index adjusting agents (light green spheres) to achieve better optical homogeneity. **b.** Boiled egg white pieces before and after incubating in PBS and 1M *N*-methylglucamine (NMG) at 37°C for 1 hour, showing that the latter can re-dissolve boiled egg white. **c.** Gross appearances of 1 mm-thick human cerebellar tissue slices after incubating in PBS, 32% iohexol solution, 25% TDE solution, 32% iohexol + 25% TDE (TI), 10% urea + 32% iohexol + 25% TDE (UTI), 20% *N*-methylglucamine + 32% iohexol + 25% TDE (NTI) at 37°C overnight. 20% *N*-methylglucamine can thus substitute 10% urea in our tissue clearing agent.

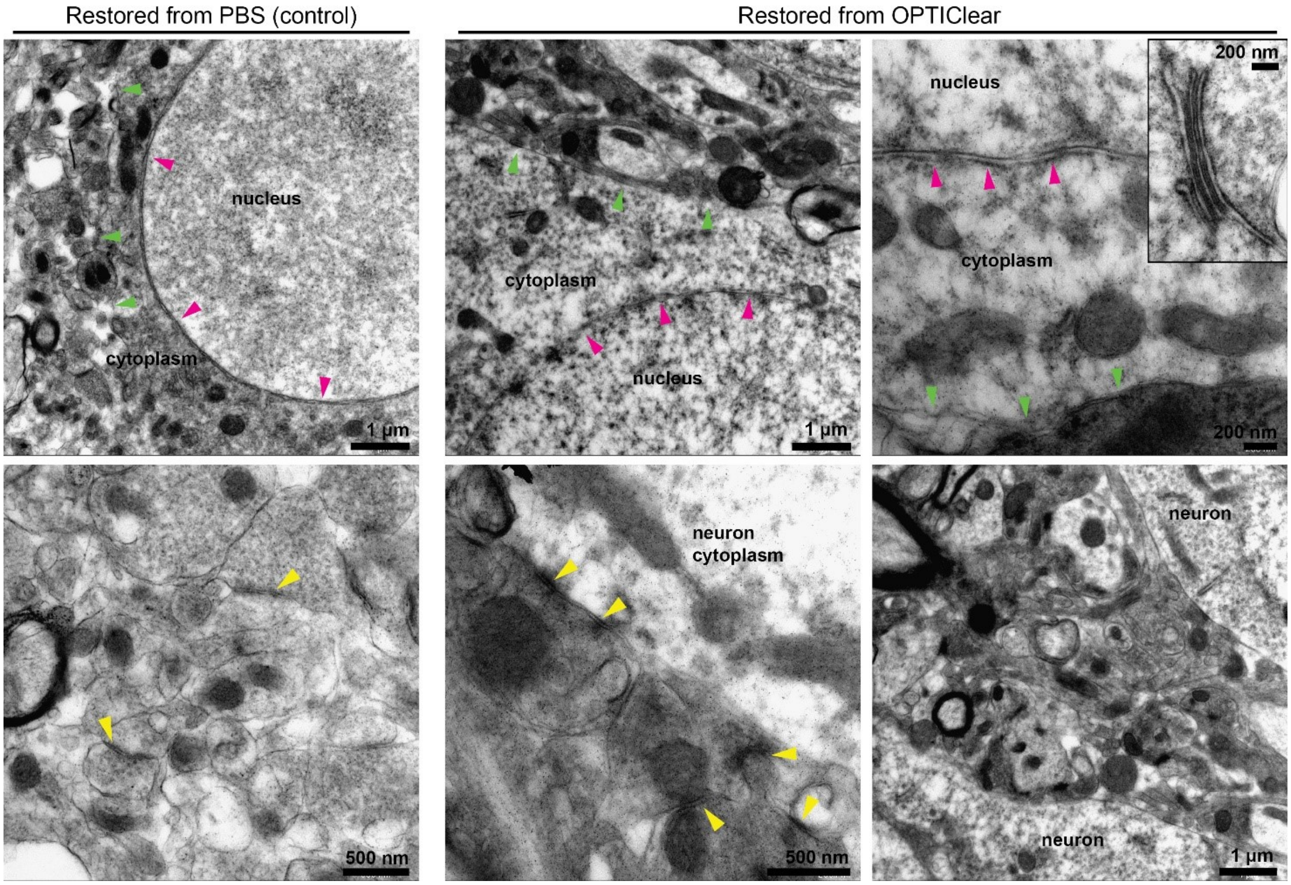

Supplementary Figure 3. Ultramicroscopic appearance of mouse brain samples restored from OPTIClear or PBS. The plasma membranes (green arrowheads) and nuclear membranes (magenta arrowheads) were well-preserved and clearly demonstrated. Synaptic structures can also be seen (yellow arrowheads). Other membranous structures such as mitochondria and Golgi apparatus can also be demonstrated (upper right corner).

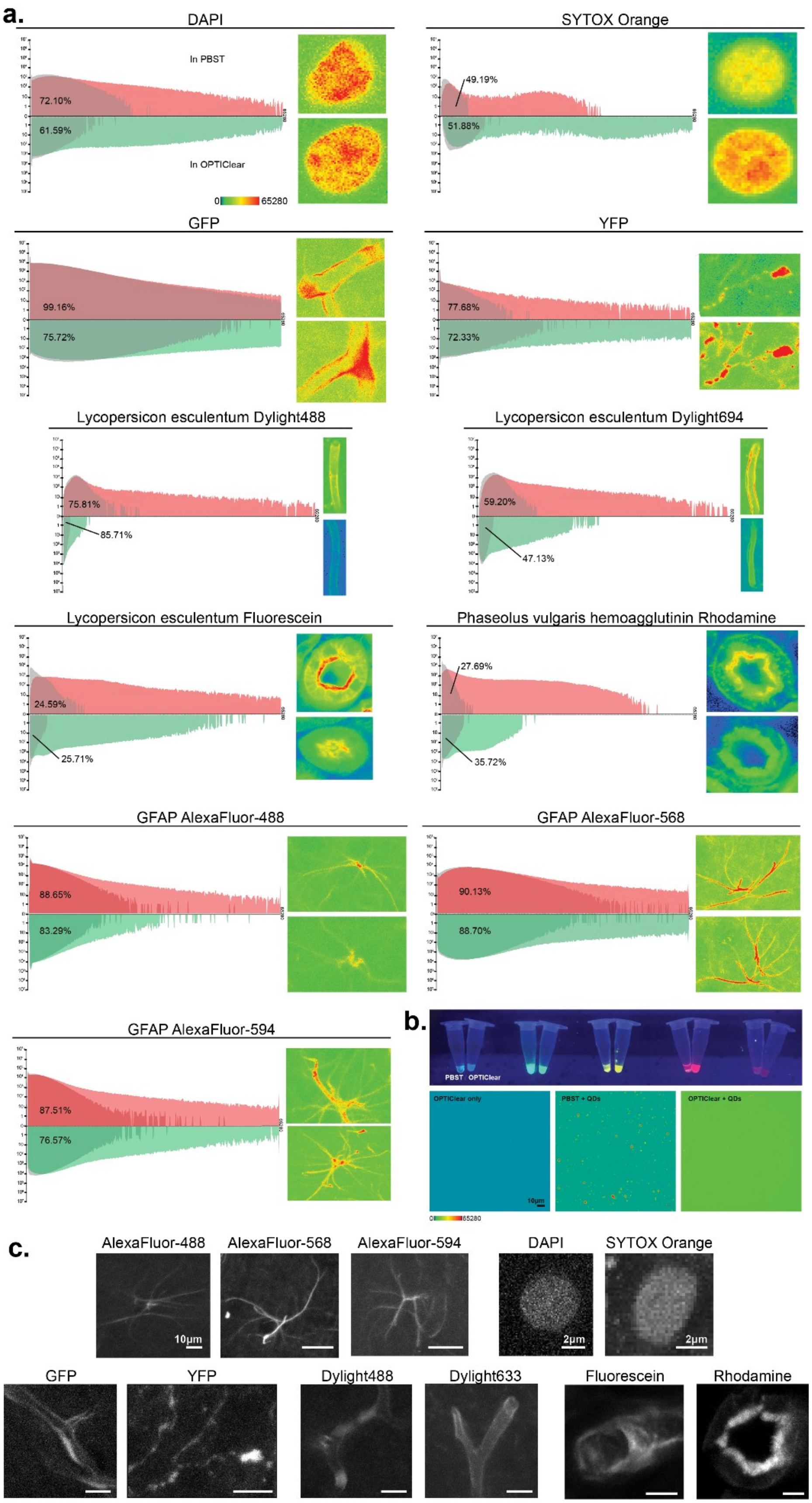

a.
DAPI
In PBST
72.10%
61.59%
In OPTIClear
0
65280
SYTOX Orange
49.19%
51.88%
GFP
99.16%
75.72%
YFP
77.68%
72.33%
Lycopersicon esculentum Dylight488
75.81%
85.71%
Lycopersicon esculentum Dylight694
59.20%
47.13%
Lycopersicon esculentum Fluorescein
24.59%
25.71%
Phaseolus vulgaris hemoagglutinin Rhodamine
27.69%
35.72%
GFAP AlexaFluor-488
88.65%
83.29%
GFAP AlexaFluor-568
90.13%
88.70%
GFAP AlexaFluor-594
87.51%
76.57%
b.
PBST
OPTIClear
OPTIClear only
PBST + QDs
OPTIClear + QDs
10µm
c.
AlexaFluor-488
AlexaFluor-568
AlexaFluor-594
DAPI
SYTOX Orange
10µm
2µm
2µm
GFP
YFP
Dylight488
Dylight633
Fluorescein
Rhodamine

Supplementary Figure 4. Testing fluorescent dye compatibility with OPTIClear. a. Mouse brain tissue was stained with various probes/fluorescent dye-conjugated reagents and imaged before and after 24 hours of incubation in OPTIClear under the same parameters. The left histograms (upper and lower, before and after OPTIClear, respectively) show the grayscale unit distributions of true signal intensities (red or green colored) and background signal intensities (grey). The percentages in each histogram states the overlapping counts between the true and background signals as a quantitative measure of image feature contrast (for details please refer to the Supplementary Methods). **b.** CdSeS/ZnS alloyed quantum dot fluorescence is preserved in OPTIClear. Upper panel: quantum dots (QD) incubated in PBST and OPTIClear at 37°C for 1 day under 302 nm ultraviolet illumination. From left to right: λ_{em} = 490 nm, 540 nm, 575 nm, 630 nm, 665 nm. Lower panel: confocal images of quantum dot (λ_{em} = 575 nm) in PBST and OPTIClear, the homogenous fluorescence observed in OPTIClear + QD indicates the QDs remained dispersed and fluorescent (ca. OPTIClear only). **c.** The same samples after 3 weeks of storage in OPTIClear at room temperatures, where the signal intensities were significantly reduced and had to be imaged using reoptimized imaging parameters. Only the stained features were cropped and shown here.

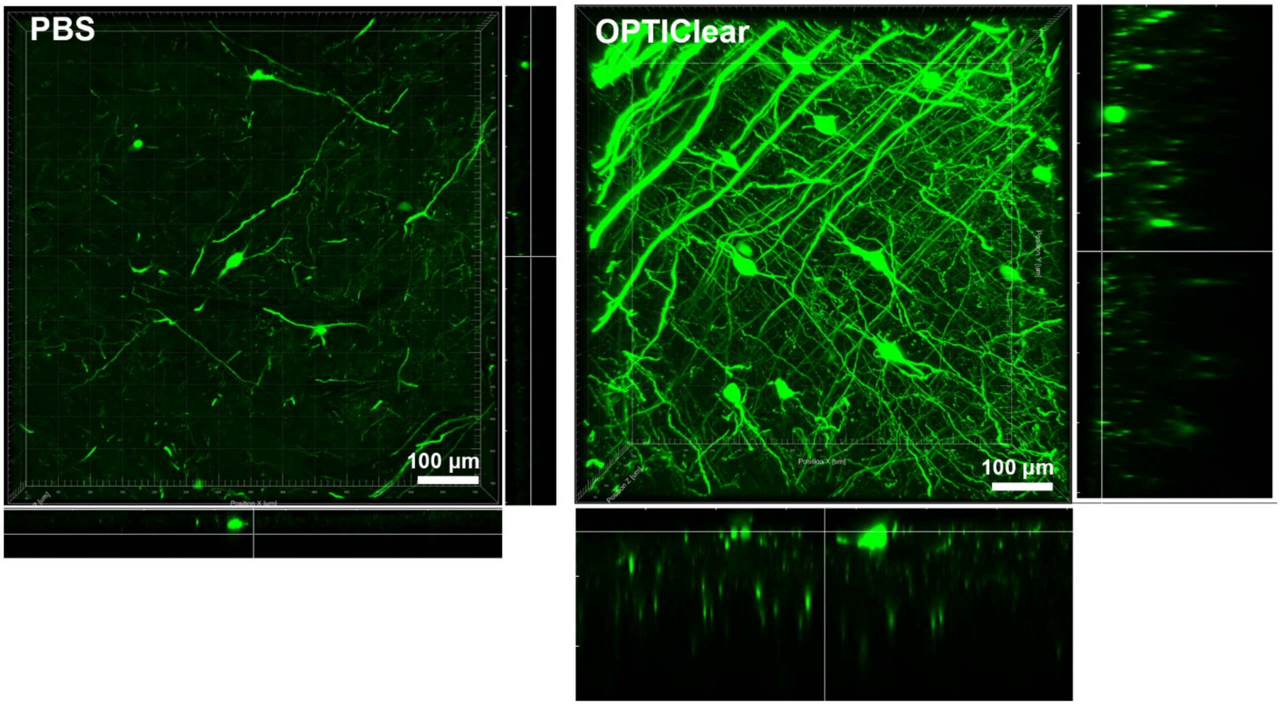

Supplementary Figure 5. Application of OPTIClear to Thy1-GFP mouse pons fixed for 1.5 years. The endogenous fluorescence is well-preserved and allowed the visualisation of structures 300 μm deep without prior tissue delipidation.

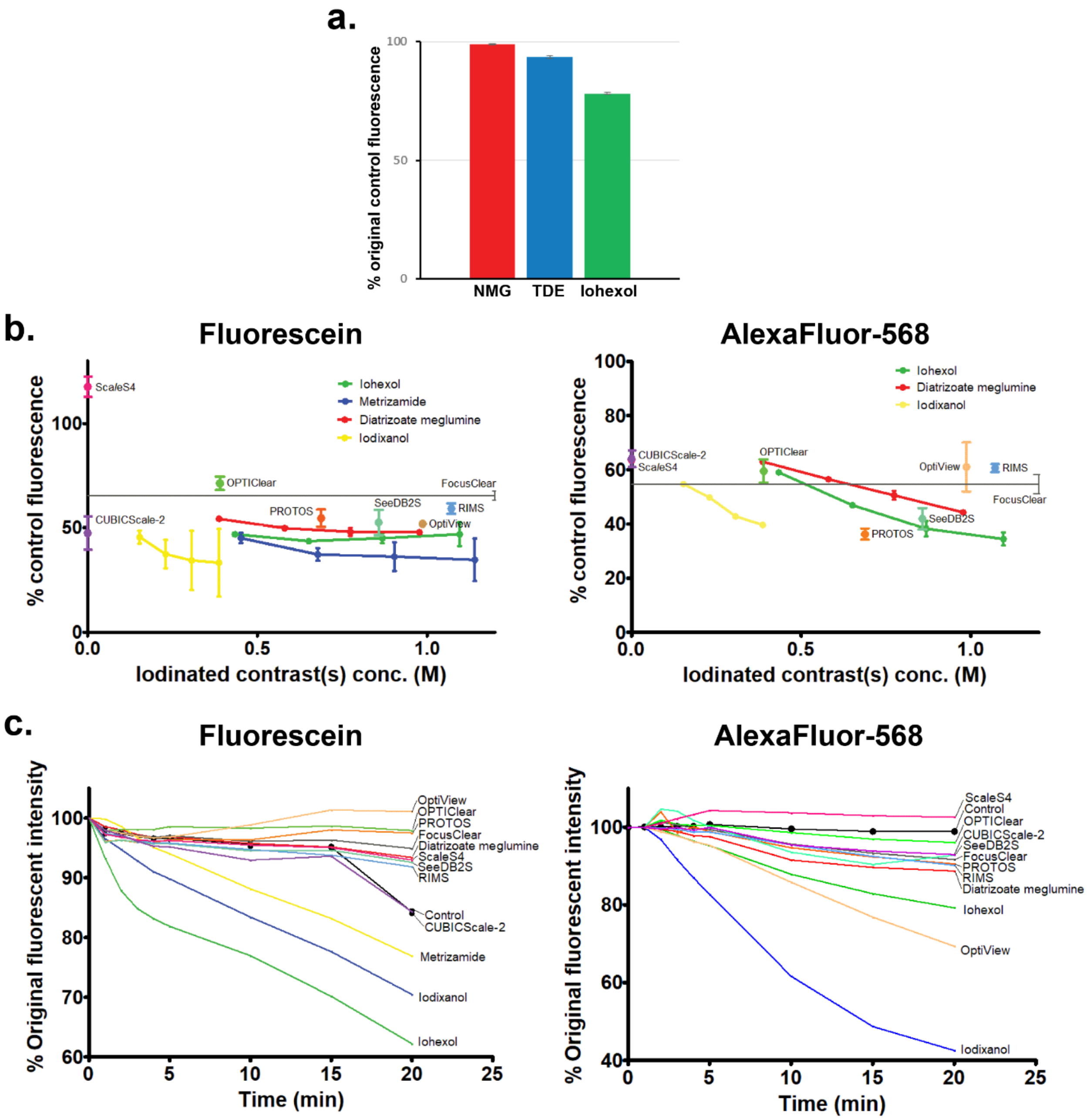

Supplementary Figure 6. Interaction of tissue clearing agents with fluorescein and AlexaFluor-568. a. Out of the three components of OPTIClear, we found 80% w/v *N*-methylglucamine (NMG) and 100% 2,2'-thiodiethanol (TDE) causes little quenching of fluorescence compared to 90% w/v iohexol, leading us to hypothesize iohexol as the component responsible for the decreased fluorescent intensity for xanthene dyes (e.g. fluorescein, AlexaFluor-488, Dylight488, and rhodamine) observed in Supplementary Figure 4. **b.** Interaction of iodinated radiocontrasts and tissue clearing agents with fluorescein (left) and AlexaFluor-568 tyramide (right), which showed that there exists a negative correlation between the concentration of iodinated contrast used and fluorophore fluorescence retained. **c.** Bleaching resistance of fluorophores in the presence of iodinated radiocontrasts or tissue clearing agents. 90% w/v iohexol, 90% w/v metrizamide, 60% w/v diatrizoate meglumine, and 60% w/v iodixanol were used for the experiment. All error bars are

standard deviations of 5 experimental replicates (except for Sca*l*eS which had 3) with 3 repeated measurements each.

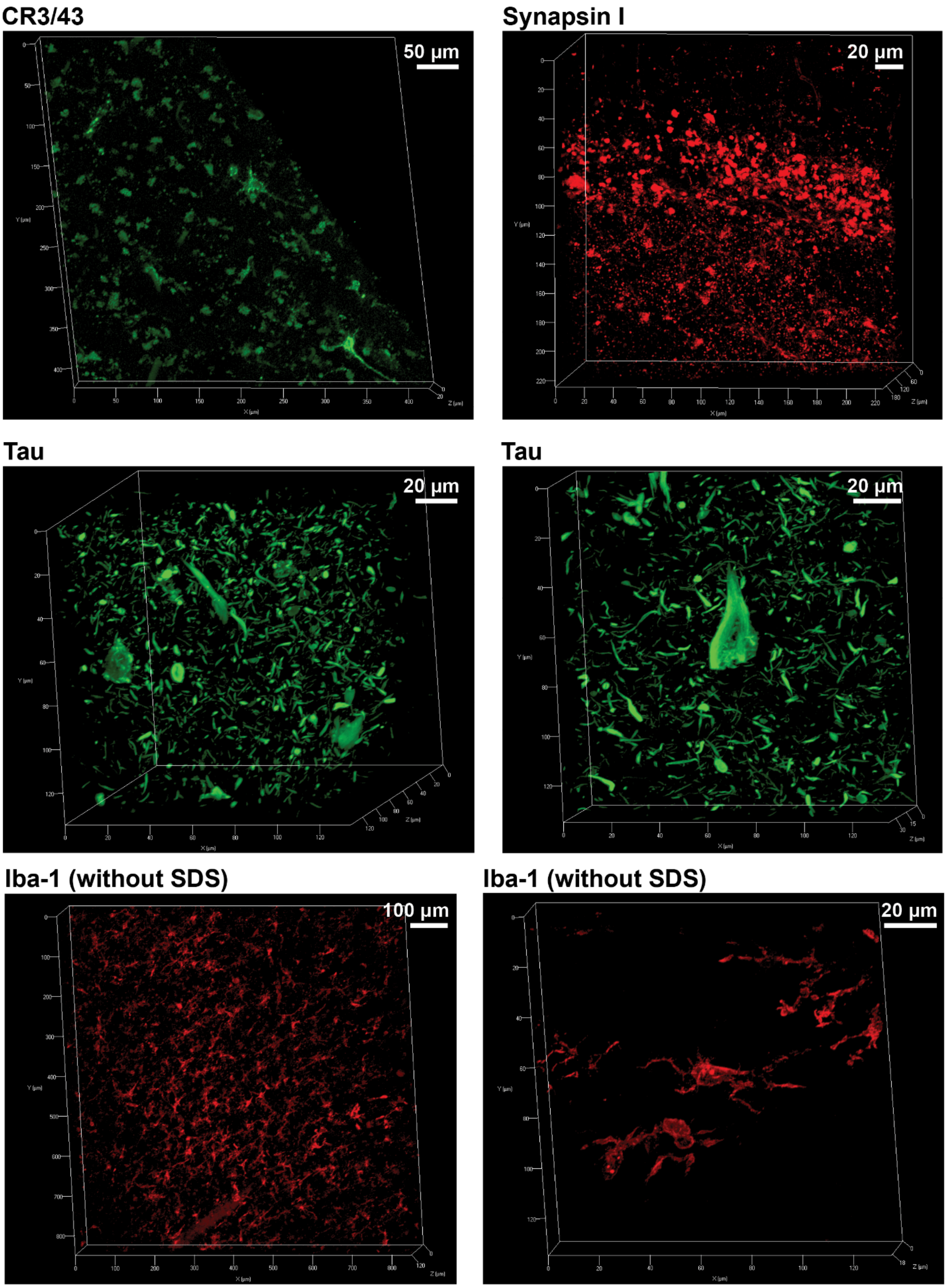

CR3/43
50 µm
Synapsin I
20 µm
Tau
20 µm
Tau
20 µm
Iba-1 (without SDS)
100 µm
Iba-1 (without SDS)
20 µm

Supplementary Figure 7. 3D immunofluorescence with next generation histology. Shown here for CR3/4, synapsin I, tau, and Iba-1.

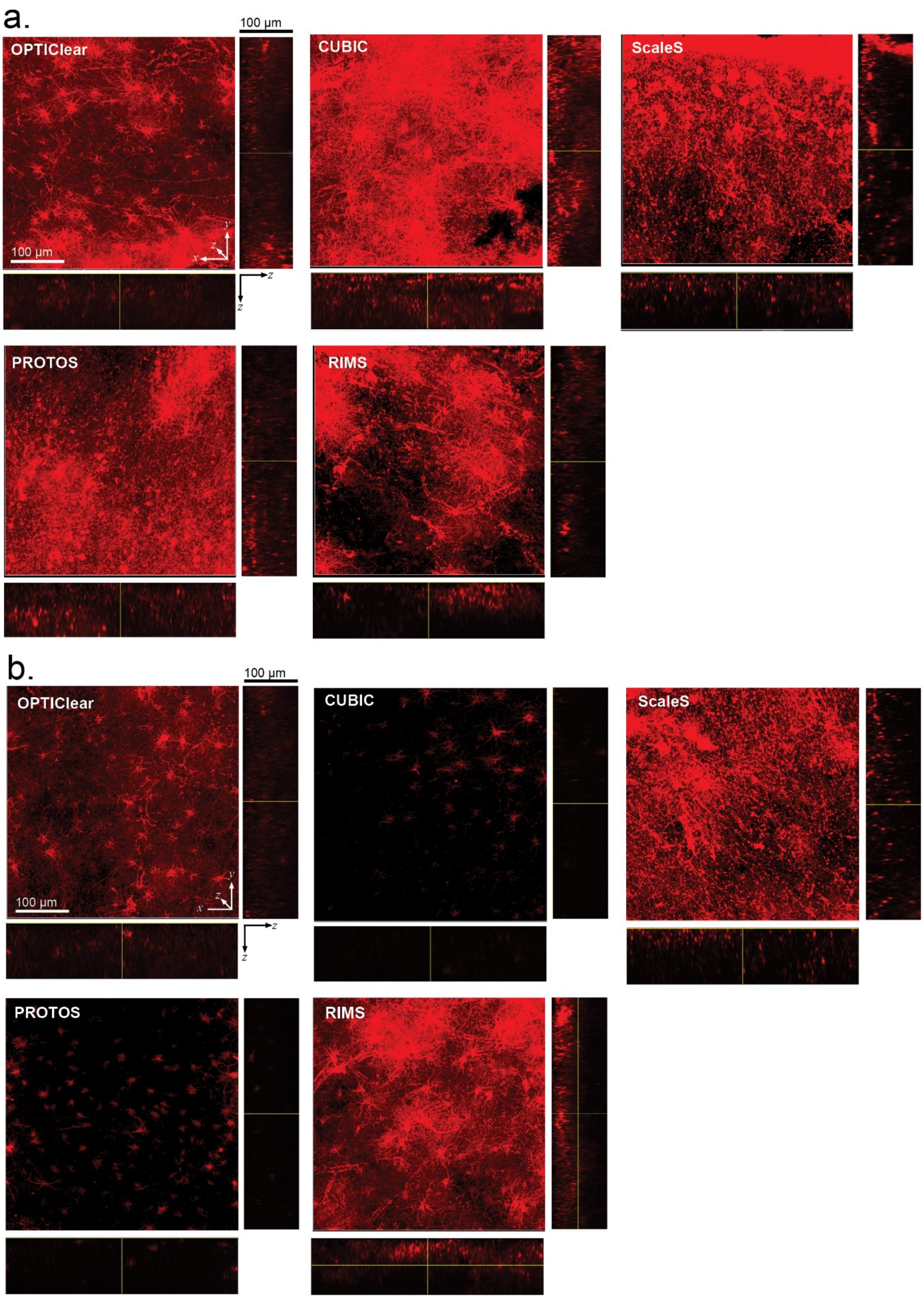
a.
100 µm
OPTIClear
CUBIC
ScaleS
100 µm
y
z
x
z
z
PROTOS
RIMS
b.
100 µm
OPTIClear
CUBIC
ScaleS
100 µm
y
z
x
z
z
PROTOS
RIMS

Supplementary Figure 8. Comparison of various tissue clearing agents in terms of preservation of immunofluorescence signals. Anti-GFAP antibodies were applied on 1 mm-thick human motor cortex slices and immersed in various RI homogenisation formulae. **a.** Stained samples after 6 hours of RI homogenisation in various formulae at 37°C. **b.** The same samples after 3 days of storage in their respective RI homogenisation solutions. Optimal parameters for confocal laser microscopy were set for individual tissues in different clearing agents. The same parameters were then applied to obtain images in the panel **b**.

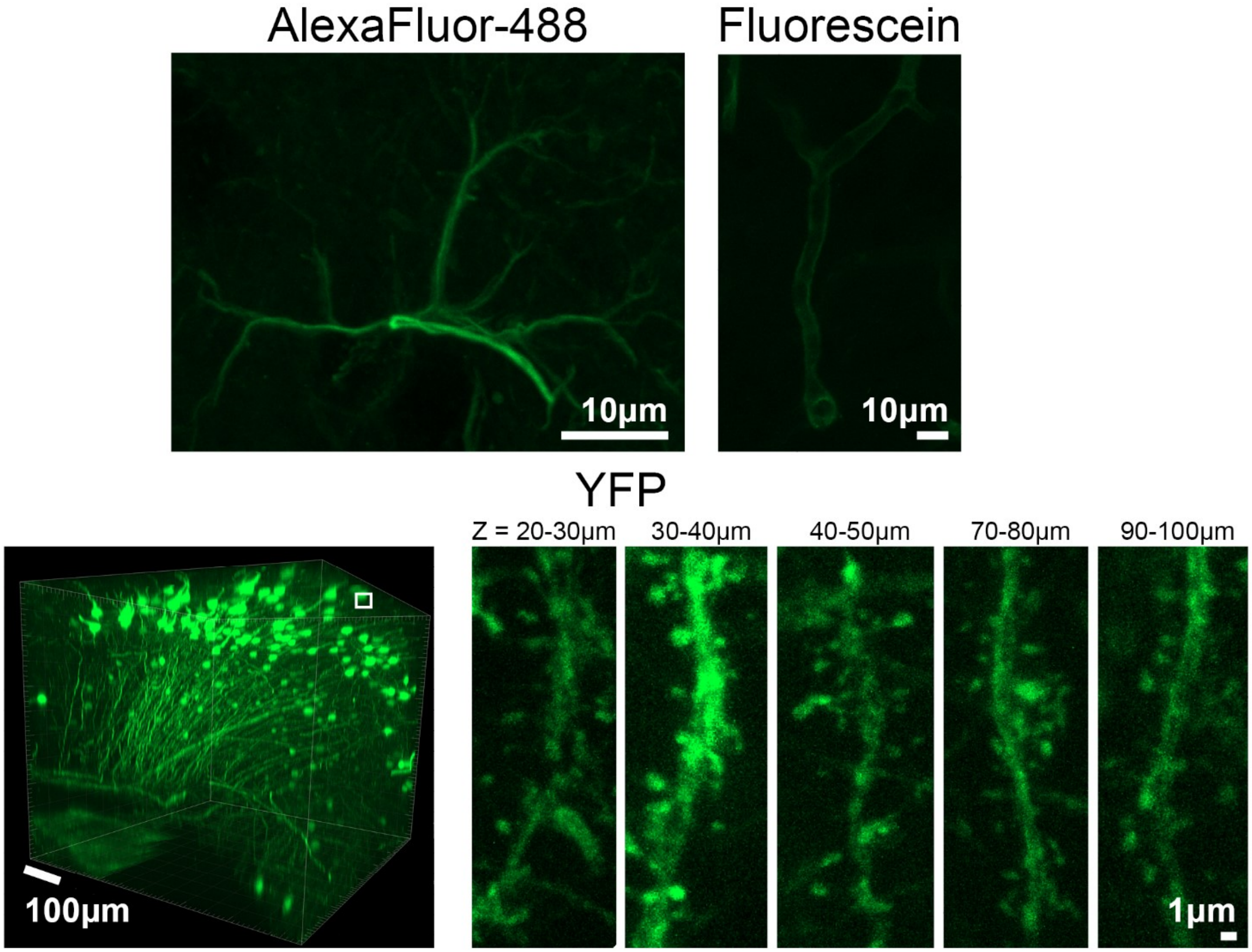

Supplementary Figure 9. Long term stability of tissue and fluorescence in OPTIClear. Non-fixed fluorescent labels (e.g. antibodies, lectins) are not as well-preserved as fixed fluorescent labels such as YFP. AlexaFluor-488: GFAP immunofluorescence in a mouse brain slice stored in OPTIClear for 740 days. Laser power 15%, digital 454 V. Fluorescein: FITC-*Lycopersicon esculentum* applied to label blood vessels in a mouse brain slice stored in OPTIClear for 645 days. Laser power 23%, digital gain 633 V. YFP: Thy1-YFP mouse brain stored in OPTIClear for 582 days. Laser power 7%, digital gain 642 V. Structures are well-preserved after long term storage in OPTIClear, as evidenced by the preservation of fine structures e.g. dendritic spines (from white boxed area of the left image).

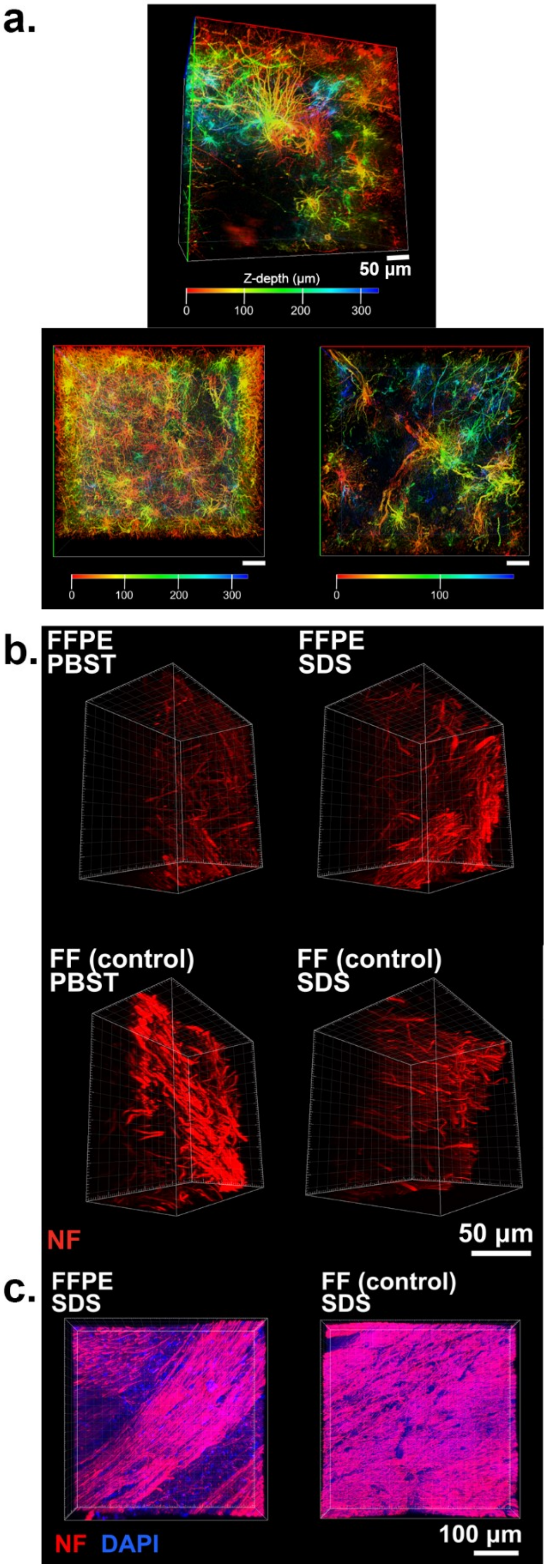

Supplementary Figure 10. Tissue clearing and 3D immunofluorescence in archived tissue. **a.** GFAP immunofluorescence for human cingulate cortex astrocytes in a sample that has been formalin-fixed for

>30 years. Related to **Figure 1b**. **b.** Comparison of immunostaining quality using anti-neurofilament (NF) antibodies on retrieved FFPE tissues with or without 4% SDS treatment. The penetration of antibodies is significantly better with 4% SDS treatment for 1 day at 55°C (indicated by “SDS”) than treatment with PBST alone (indicated by “PBST”). Note that the immunostaining quality and penetration of antibodies for SDS-treated, retrieved-FFPE tissue is comparable to the control, conventionally formalin-fixed (FF) tissues under the same SDS-treatment and immunostaining conditions. **c.** A further comparison on another batch of retrieved FFPE pons with a control, formalin-fixed (FF) tissue. Nuclear staining quality with DAPI is also similar for both cases.

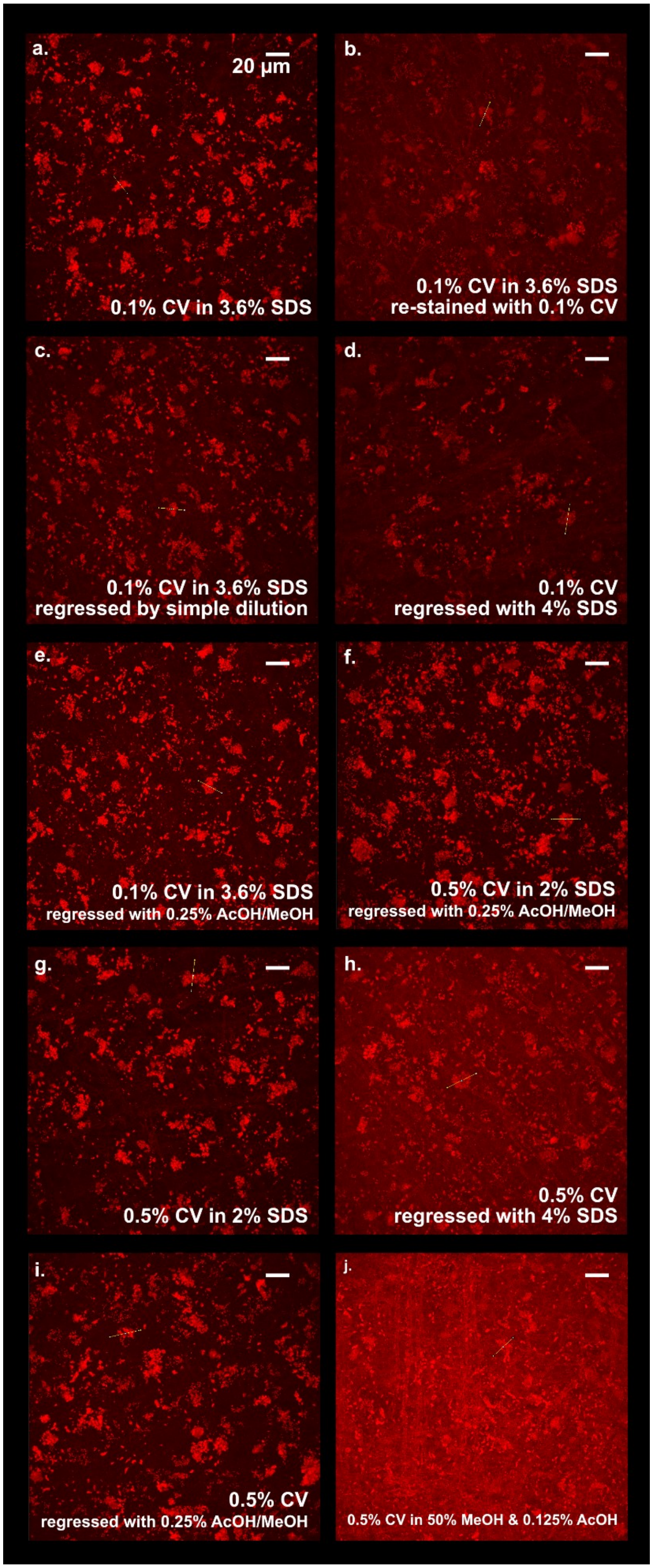
a.
20 µm
0.1% CV in 3.6% SDS
b.
0.1% CV in 3.6% SDS
re-stained with 0.1% CV
c.
0.1% CV in 3.6% SDS
regressed by simple dilution
d.
0.1% CV
regressed with 4% SDS
e.
0.1% CV in 3.6% SDS
regressed with 0.25% AcOH/MeOH
f.
0.5% CV in 2% SDS
regressed with 0.25% AcOH/MeOH
g.
0.5% CV in 2% SDS
h.
0.5% CV
regressed with 4% SDS
i.
0.5% CV
regressed with 0.25% AcOH/MeOH
j.
0.5% CV in 50% MeOH & 0.125% AcOH

Supplementary Figure 11. Development of an optimized 3D Nissl staining protocol. Various staining conditions and regression conditions were tested and compared. All tissues used were 1 mm-thick pieces of human motor cortex (around layer V) from a healthy control. As seen from the optimisation results tissue staining with 0.1% cresyl violet in 3.6% SDS followed by regression in 0.25% acetic acid in methanol (**e.**) gives the best signal contrast and minimal nonspecific neuropil staining, which was confirmed with quantification of signal intensities measured along the yellow lines within the images (data not shown). Scale bars: 20 µm. AcOH: acetic acid; CV: cresyl violet; MeOH: methanol; SDS: sodium dodecyl sulphate.

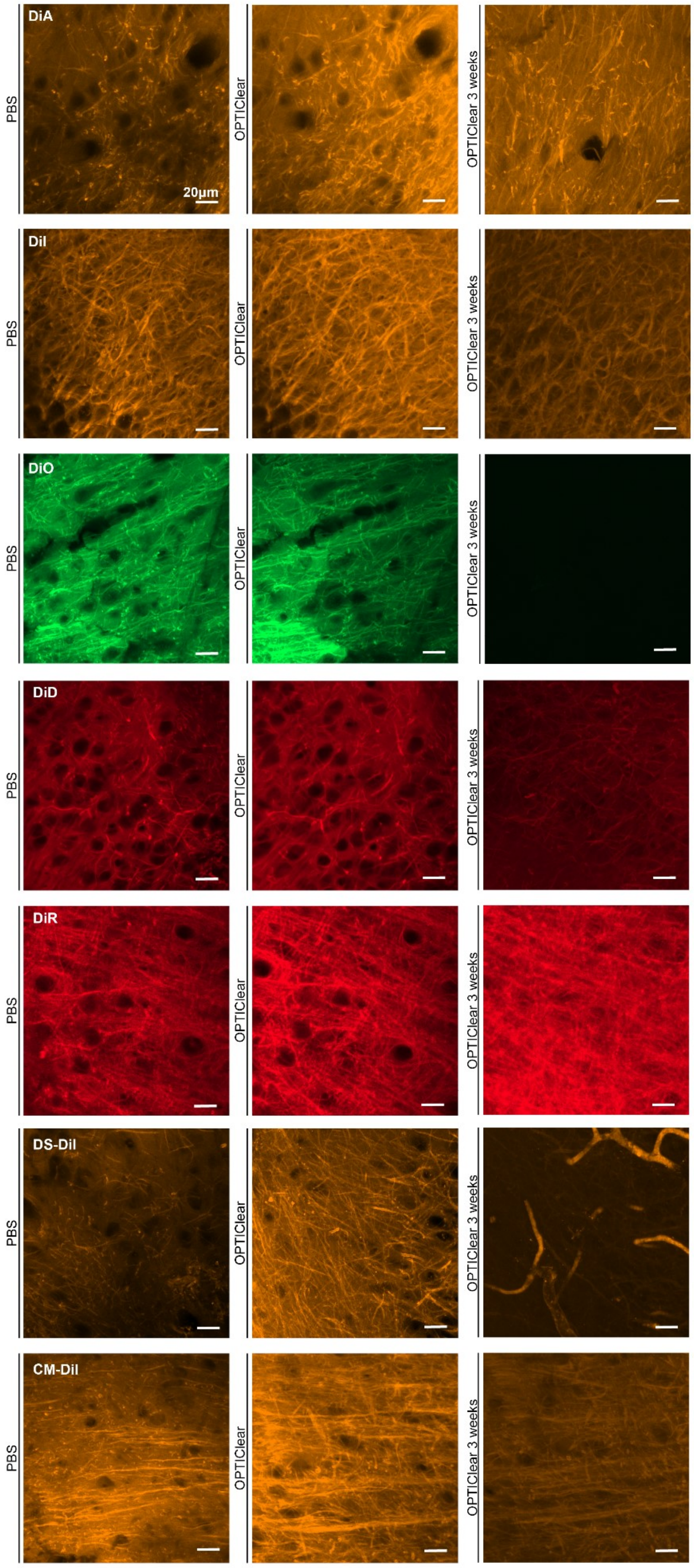
DiA
DiI
DiO
DiD
DiR
DS-DiI
CM-DiI
PBS
OPTIClear
OPTIClear 3 weeks
20µm

Supplementary Figure 12. Lipophilic tracer dyes compatible with OPTIClear. Three rounds of imaging were performed in the same tissue samples immediately before OPTIClear (left column), after overnight OPTIClear incubation (middle column), and after 3 weeks of storage in OPTIClear at room temperature (right column). Scale bars: 20 µm.

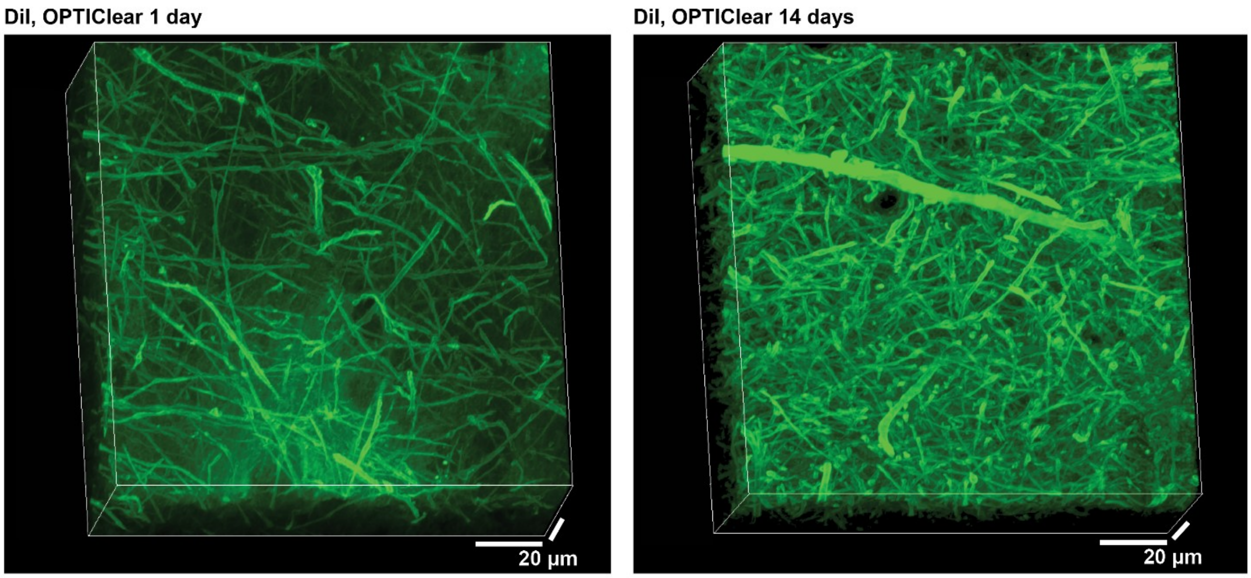

Supplementary Figure 13. Long term stability of DiI tracing in human brain tissue in OPTIClear. The DiI tracings in human temporal cortex were not disrupted after storing in OPTIClear for 14 days at room temperature.

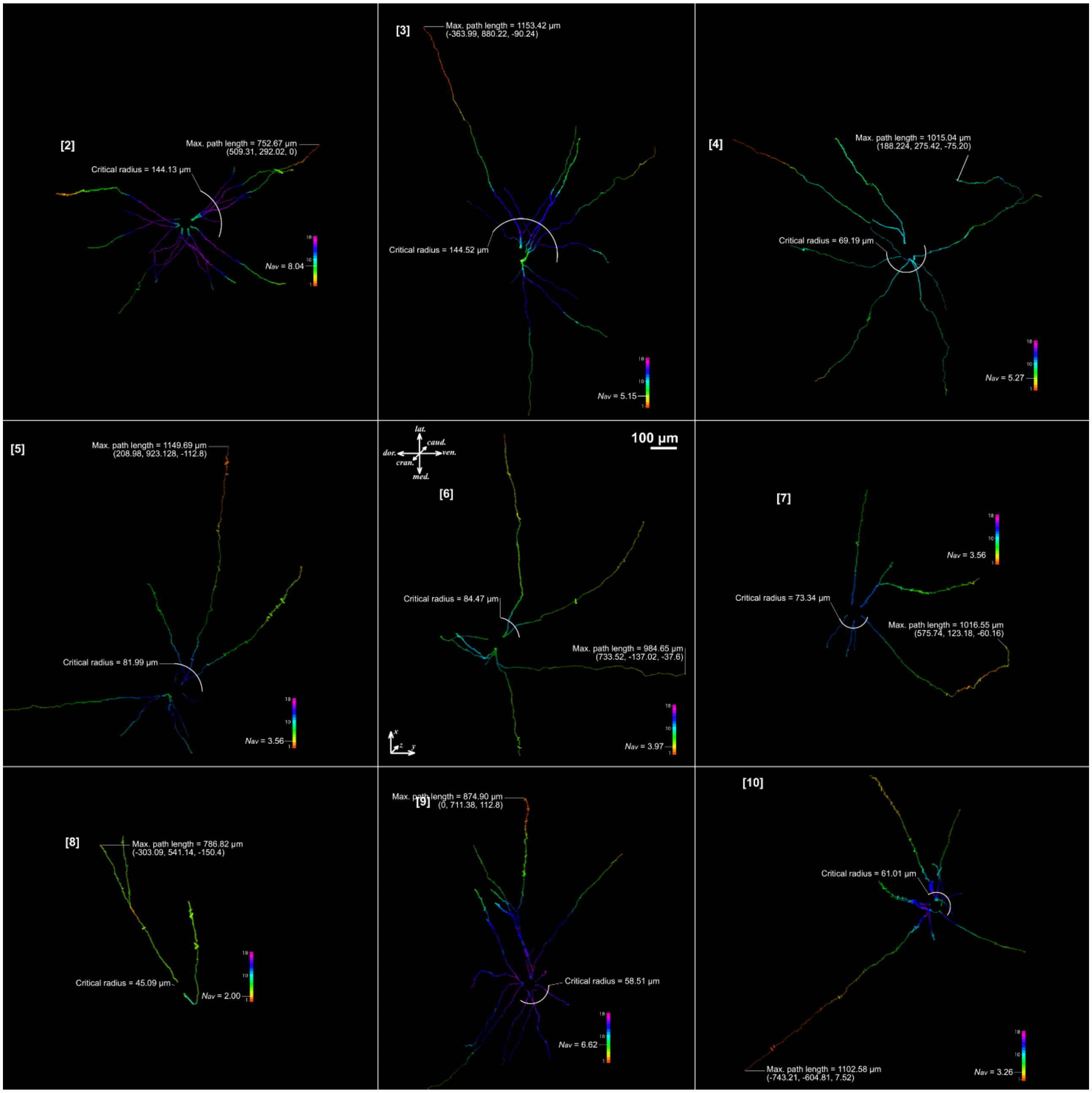

Supplementary Figure 14. Morphometric parameters of TH-positive neurons traced in the reticular formation in Figure 4b. The cartesian coordinates (in µm) were labelled for the longest path length with the soma marked as the origin, from which the Euclidean distance can be calculated. The number of intersections was colour-coded. N*av* is the average number of intersections of the entire arborization on Sholl analysis, and is computed by dividing the area under the fitted polynomial function by the maximal radial extent of the neuronal fibers. The critical radius is the radial distance from soma where the maximum number of intersections occurs.

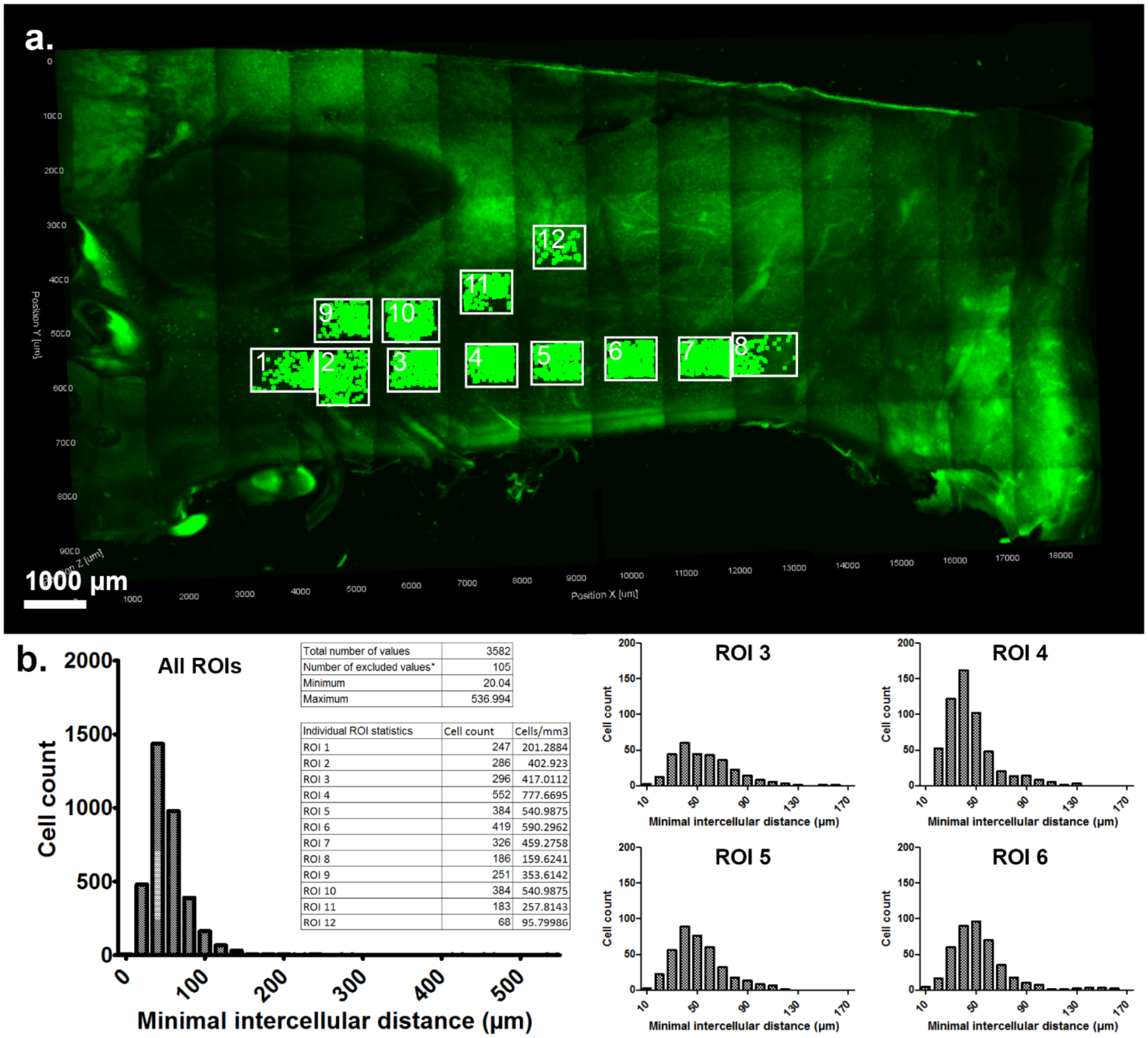

Total number of values	3582
Number of excluded values*	105
Minimum	20.04
Maximum	536.994

Individual ROI statistics	Cell count	Cells/mm3
ROI 1	247	201.2884
ROI 2	286	402.923
ROI 3	296	417.0112
ROI 4	552	777.6695
ROI 5	384	540.9875
ROI 6	419	590.2962
ROI 7	326	459.2758
ROI 8	186	159.6241
ROI 9	251	353.6142
ROI 10	384	540.9875
ROI 11	183	257.8143
ROI 12	68	95.79986

Supplementary Figure 15. Additional statistics for the magnocellular neurons surveyed in Figure 5. Regions of interest (ROIs) were marked and numbered in white boxes in **a. b.** The left histogram shows pooled data from all ROIs, the histograms from ROIs 3-6 were presented on the right.

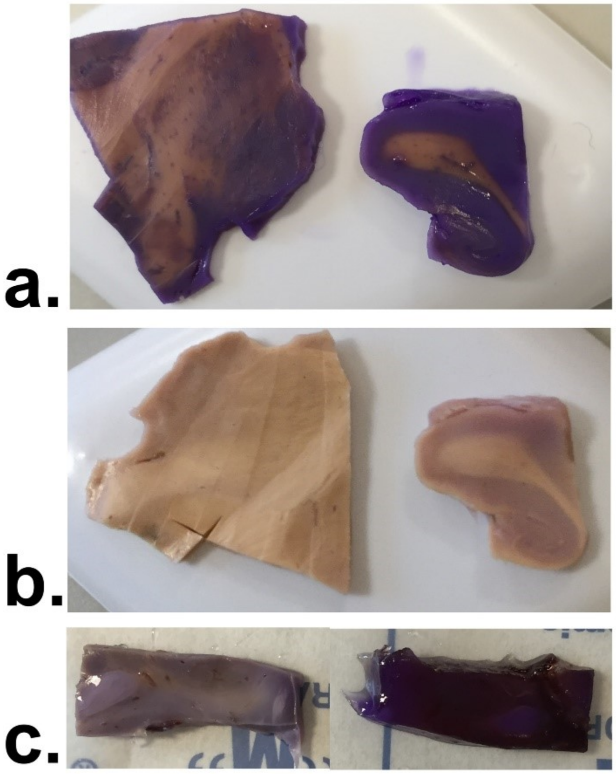

Supplementary Figure 16. Gross appearances of human tissue samples during the 3D Nissl staining procedure. In **a.** and **b.**, a piece of thalamus coronal section and hippocampus was shown on the left and right, respectively; while a piece of basal forebrain coronal section featuring the nucleus basalis of Meynert (same as that as in **Figure 5a**, inset) was shown in **c.** After initial staining in 0.1% cresyl violet/3.6% SDS (**a.**), the grey matter is stained intensely purple while the white matter is virtually unstained. Following regression in 0.25% acetic acid in methanol (**b.**), the grey-white matter differentiation is almost lost. **c.** After OPTIClearing, the tissue looks paradoxically darker as the stained-tissue can now be visualised even though they were physically buried under the surface of the tissue, which is an indication of successful tissue clearing.

Supplementary Tables

Supplementary Table 1. Comparison of existing aqueous-based tissue clearing methods for a hypothetical 3-week formalin-fixed, 2 mm-thick human brain tissue with the use of immunostaining.

Method	Current method	CLARITY	SWITCH	FASTClear (aqueous-based)	AbSca*le*S
**Step 1**	Formalin fixation‡				
**Step 2**	• SDS treatment*	• Wash in PBS<br>• Acrylamide infiltration and polymerization<br>• Wash in PBST<br>• SDS treatment ^	• Wash in buffer (pH3)<br>• Glutaraldehyde fixation<br>• Wash in PBS<br>• Aldehyde quenching	• Wash in PBS<br>• SDS treatment^	• Wash in Sca*l*eS0<br>• Permeabilization steps 1, 2 and 3
**Step 3**	Antibody incubation<br>Primary antibody → wash → secondary antibody → wash				
**Step 4**	N/A	N/A	N/A	N/A	Re-fixation → wash
**Step 5**	Refractive index homogenisation				

‡ Usually pre-fixed for human tissues.

* Can be partial (at least 1 day) to complete (up to 3.5 months).

^ Must be complete (average 40 days).

Supplementary Table 2. Effects of different chemicals on boiled egg whites at a concentration of 0.5M.

Completely dissolved	Partially dissolved	No observable changes
Urea	SDS	Propylene glycol
Ethylenediamine	Guanidinium chloride	Ethylene glycol
	N-methylglucamine	Glycerol
		Putrescine hydrochloride
		N,N,N′,N′-Tetramethylethylenediamine
		Ethylenediaminetetraacetic acid
		Choline chloride
		Hydrazine hydrate
		Hydroxylammonium chloride
		Triethanolamine
		Triton X-100
		N-lauroylsarcosine sodium
		N,N,N′,N′-Tetrakis(2-hydroxypropyl)ethylenediamine
		N-Acetyl-4-hydroxyproline

Supplementary Table 3. Tissue processing procedures for individual samples displayed in the figures of this paper.

**Figure number**	**Final Neuro-pathological Diagnosis**	**Formalin -fixation duration**	**FFPE retrieval (and duration of archival)**	**Duration of treatment with 4% SDS solution at 55ºC**	**Immunostaining strategy (for both primary and secondary antibodies)**	**Duration of OPTIClearing at 37ºC**
Fig. 1b	CTE	Approx. 40 years	N/A	4 months	1 week with alternate day supplementation of antibodies at 1:100 dilution	Overnight
Fig. 1c	PD	3 weeks	Yes (3 years)	5 days	1 day at 1:100 dilution	3 hours
Fig. 2a.	PD	3 years	N/A	10 days	None	6 hours
Fig. 2b.	CTE	Approx. 30 years	N/A	3 weeks	2 days at 1:100 dilution with lectin (1:100) added during secondary antibody incubation	Overnight
Fig. 2c-e.	PD	3 years	N/A	None	None	Overnight
Fig. 3a.	PD	3 years	N/A	7 days	2 days with daily supplementation of antibodies at 1:100	Overnight
Fig. 3b.	Normal	1 week	N/A	3.5 months	10 days with alternate day supplementation of antibodies at 1:100	3 hours
Fig. 4	Normal	1 week	N/A	3.5 months	2 days at an initial 1:100 dilution, followed by re-staining* at initial dilution of 1:50 for 2 days with 2 times supplementation at 1:50 for 2 days each	6 hours
Fig. 5	Normal	3 weeks	N/A	3 days	None	Overnight

* This sample has been OPTICleared after the first staining session only to found that the amount of antibodies used was insufficient, and therefore it was restored by washing in PBST and restained.

Abbreviations: CTE: Chronic Traumatic Encephalopathy; DLB: Dementia with Lewy bodies; PD: Parkinson's disease

Supplementary Table 4. Compatibilities of reagents with OPTIClear.

Reagent (Catalog no.)	After 24h of OPTIClear (compared with PBST)
Donkey anti-Rabbit IgG, AlexaFluor 488 conjugate (Invitrogen A21202)	Decreased fluorescence intensity
Donkey anti-Rabbit IgG, AlexaFluor 568 conjugate (Invitrogen A10042)	Retained fluorescence intensity
Goat anti-Rabbit IgG, AlexaFluor 594 conjugate (Invitrogen A11012)	Retained fluorescence intensity
DAPI (Sigma D9542-10MG)	Slightly decreased fluorescence intensity
SYTOX^{TM} Orange (Invitrogen S11368)	Increased fluorescence intensity
EGFP (EGFP-SD transgenic rat)	Retained fluorescence intensity
YFP (Thy1-YFP transgenic mouse)	Increased fluorescence intensity
Lycopersicon esculentum lectin, Dylight488 conjugated (Vector laboratories DL-1174)	Greatly decreased fluorescence intensity
Lycopersicon esculentum lectin, Dylight694 conjugated (Vector laboratories DL-1178)	Decreased fluorescence intensity
Lycopersicon esculentum lectin, Fluorescein-conjugated (Vector laboratories FL-1171)	Decreased fluorescence intensity
Phaseolus vulgaris hemoagglutinin, Rhodamine-conjugated (Vector laboratories RL-1122)	Greatly decreased fluorescence intensity
DiI (Lipophilic sampler kit, Invitrogen L7781)	Retained signal intensity
DiA (Lipophilic sampler kit, Invitrogen L7781)	Retained signal intensity
DiO (Lipophilic sampler kit, Invitrogen L7781)	Slightly decreased signal intensity
DiD (Lipophilic sampler kit, Invitrogen L7781)	Retained signal intensity
DiR (Lipophilic sampler kit, Invitrogen L7781)	Retained signal intensity
CM-DiI (Lipophilic sampler kit, Invitrogen L7781)	Retained signal intensity
$DiIC_{18}(3)$-DS (Lipophilic sampler kit, Invitrogen L7781)	Retained signal intensity
5,5'-Ph_2-$DiIC_{18}(3)$ (Lipophilic sampler kit, Invitrogen L7781)	Loss of signal
SP-$DiIC_{18}(3)$ (Lipophilic sampler kit, Invitrogen L7781)	Loss of signal
SP-$DiOC_{18}(3)$ (Lipophilic sampler kit, Invitrogen L7781)	Loss of signal
CdSeS/ZnS alloy quantum dots (Sigma 753866, 754226, 753890, 753882, 753874)	Retained fluorescence intensity

Supplementary methods

Derivation of OPTIClear

The available aqueous-based tissue clearing agents rely on a single chemical with high molar refractive index used at high concentrations, which presumably works by raising different compartments' RI to an optimal value to achieve optical homogeneity. Identifying the ideal chemical could be difficult, as the tissue clearing efficacy of a chemical is governed by three factors: (1) the RI-adjusting property, which depends on the electronic configuration of the molecule; (2) its penetration and distribution, determined by its chemical structure and partition coefficient; and (3) its reactivity governed by the choice of functional groups. Furthermore, for practical purposes, it is important to strike an intricate balance between these factors, such that the tissue clearing reagent would have low viscosity, osmolarity and cost.

While the ideal "tissue clearing chemical" is difficult to design or identify, we reasoned that optical homogeneity may be achievable by using multiple chemicals, where each of them could be responsible for adjusting the RI of specific tissue compartments. Based on the Lorentz-Lorenz equation, we chose chemicals with a large delocalized electron field that would impart a greater refractive index increase in solution even at low concentrations, such as those containing aromatic rings, iodine atoms, and sulfur atoms. Hydroxyl groups are also essential for water solubility and tunable RI in correlation with the concentration. Meanwhile, we noted that stability is important as chemicals with high polarizability tend to be strong nucleophiles or reducing agents. Finally, the structure of the chemicals governs their solubility in different solvents, which not only affects their permeability in non-delipidated tissues, but also affect their partition coefficients between aqueous and relatively hydrophobic compartments such as lipid membranes and lipofuchsin granules, which would be essential to our idea of constructing a multi-component tissue clearing agent containing compartment-selective refractive index-adjusting agents. Based on the above criteria, structural analysis, the list of reagents that has been used as tissue clearing agents and our experience with their tissue clearing efficacies, we chose iohexol and 2,2'-thiodiethanol as the RI adjusting agent for hydrophilic and hydrophobic compartments in tissues, respectively. Consistent with our idea of compartment-selective RI adjustment using these two chemicals with different partition coefficients, they demonstrated a synergistic effect in tissue clearing (**Supplementary Figure 1**). We explored other candidates and found that metrizamide can be used in place of iohexol (**Supplementary Figure 1**), but did not perform well in the tissue setting, presumably due to its poor penetrating ability; while TDE significantly out-performed 1-thioglycerol and glycerol (**Supplementary Figure 1**), which may be explained by their lower partition coefficients and molar refractive indices.

Denaturants are an essential component in aqueous-based tissue clearing agents, which presumably opens up spaces within the dense proteinaceous cytosol to allow adequate penetration and homogeneous distribution of refractive index adjusting chemicals (**Supplementary Figure 2**). However, strong denaturants disrupt protein-protein interactions and lipid membranes, leading to incompatibility with immunohistochemistry and lipophilic tracers, respectively. To screen for a milder denaturing agent, we used boiled egg white as a platform and discovered that *N*-methylglucamine and ethylenediamine could re-dissolve boiled egg white (**Supplementary Figure 2, Supplementary Table 2**). Indeed, the use of 20% *N*-methylglucamine in place of 10% urea produced similar tissue clearing results in human tissues

(**Supplementary Figure 2**). Ethylenediamine was not used due to its reactivity towards formaldimine linkages and its hazardous nature.

Boiled egg white preparation and screening of denaturants

A piece of boiled egg white was dissected out, and cut into 1mm-thick discs with 5mm-diameter. It was then added into eppendorfs containing 0.5M of the candidate chemicals and incubated at 37°C for 1 hour, after which the mixture was observed for any changes.

Whole brain homogenate preparation

Freshly dissected mouse brains that have been perfusion-fixed and post-fixed in 4% paraformaldehyde at room temperature were mechanically meshed into a paste, after the addition of 10ml PBS per brain, the mixture was sonicated in a 50ml Falcon tube. The homogenate was stored at 4°C before use.

Absorbance measurement with spectrophotometer

Candidate chemical(s) and brain homogenates were mixed in various combinations in a 96-well plate, which was incubated at 37°C overnight. The absorbance of the incubated mixture was then read out at various wavelengths using a Perkin Elmer Victor^{3} 1420 Multilabel Counter spectrophotometer. In all experiments, pure water and brain homogenate mixed with equal volumes of water were included in two separate wells as calibrating controls.

Post-OPTIClear transmission electron microscopy

Samples were immersed in OPTIClear or 1x PBS (control) at 37°C for 3 days, which were then restored by washing in 1x PBS at 37°C for 1 day. The restored samples were then processed according to the standard procedures provided by the Electron Microscopy Unit of the University of Hong Kong (http://www.emunit.hku.hk/documents/SamplePreparationTechnique.pdf, page 1). Briefly, the samples were post-fixed with 2.5% glutaraldehyde in 0.1M sodium cacodylate buffer overnight at 4°C. The next day, the fixed samples were transferred to a solution containing 0.1M sucrose in 0.1M sodium cacodylate buffer, and incubated overnight at 4°C. The samples were then washed three times in 0.1M sodium cacodylate buffer, second-fixed with 1% osmium tetroxide in 0.1M sodium cacodylate buffer for 1 hour at room temperature, and washed clean in 0.1M sodium cacodylate buffer. The osmium-treated samples were then dehydrated with a series of 50%, 70%, 90%, 100% ethanol, and finally with propylene oxide. A 1:1 mixture of epoxy resin and propylene oxide was then added to infiltrate the blocks at 37°C overnight. The next day the blocks were incubated in pure epoxy resin at 37°C overnight, and allowed to polymerize at 65°C for at least 2 hours. Semi-thin and ultra-thin sections were obtained using a Lecia Ultracut UCT Ultramicrotomy and collected on water. Sections were stained with uranyl acetate and lead (II) acetate, and visualized using a Philips CM100 Transmission Electron Microscope.

Testing for dye compatibility in OPTIClear

All incubations were performed at 37°C unless otherwise specified. 1 mm-thick mouse brain slices and rat kidney slices were treated in 4% SBC at 37°C for 3 days, washed in PBST for 1 day, and stained in the respective testing solutions (Supplementary Table 4) at 1:100 dilutions in PBST at 37°C for 2 days. The stained samples were washed in PBST for 1 day, and stained with secondary antibodies at 1:100 dilutions in PBST for 2 days if necessary. For GFP and YFP, EGFP-SD transgenic rat and Thy1-YFP mouse brain tissues were used. For lipophilic tracer dyes, a tiny incision was made in the brain tissue and a

crystal of the dye was embedded within, the samples were then incubated in PBS at 37°C for 5 days. The samples (except for those stained with lipophilic tracers) were washed in PBST for 1 day and imaged immediately, the same samples were then incubated in OPTIClear at 37°C for 24 hours and another round of imaging was performed. All imaging parameters were kept the same for each sample with no post-imaging contrast/brightness adjustments. To quantify the changes in image contrasts and true signal intensities after incubation in OPTIClear, and to avoid biases from background signals (e.g. autofluorescence), staining density, antibody penetration, and light scattering in PBST controls, we only imaged the most superficial part of the tissue slices. We then manually selected the relevant features of staining (e.g. an astrocyte, blood vessel, or nucleus) in regions of interest (ROI) for 'true' signals, and sampled other non-stained area for background signals with the same ROI area. 6 – 9 ROIs were used for each around of imaging to ensure adequate coverage of all features in the image. The sampled true and background signals (in grayscale unit counts) were then plotted in a histogram, where the overlapping area represent the ambiguous signal counts (i.e. a signal that cannot be determined to be 'true' or background in origin given its intensity). The overlapping area divided by the total true signal counts would therefore be negatively correlated with the image contrast of the feature concerned, which is expressed in percentages in Supplementary Figure 4.

After the first two rounds of imaging, the samples were then stored in OPTIClear at room temperature for 3 weeks, after which a third round of imaging was performed, with re-optimized imaging parameters in view of the diminished signal intensity after prolonged storage at room temperature.

For quantum dots, 1 µl of the quantum dots stock solution (CdSeS/ZnS alloyed quantum dots COOH functionalised, 6 nm diameter, 1 mg/ml (Sigma 753866, 754226, 753890, 753882, 753874)) was mixed with 10 µl of PBST or OPTIClear and incubated at 37°C for 24 hours, before observing for fluorescence under 302 nm ultraviolet light and imaging in a glass slide using a confocal microscope under 305 nm laser excitation.

Testing for fluorescence stability in various tissue clearing solutions and iodinated contrast agents

Stock solutions of 90% w/v iohexol (1.096 M), 90% w/v metrizamide (1.141 M), 60% w/v diatrizoate meglumine (0.977 M), and 60% w/v iodixanol (0.387 M) were made and serially diluted with distilled water; tissue clearing solutions were prepared according to the instructions given in the literature. 1 µl of fluorescein sodium (50 mM) or AlexaFluor-568 tyramide (Invitrogen B40956, dissolved in 300 µl of distilled water) solution was then separately added to 100 µl of the above-prepared solutions. The fluorescence intensities of the dyes were then measured with a Perkin Elmer Victor^{3} 1420 Multilabel Counter spectrofluorophotometer. Excitation/emission wavelengths at 485/535 nm and 570/600 nm were used for fluorescein and AlexaFluor-568, respectively. The experiment was repeated 5 times (except for Sca*l*eS4 which was repeated 3 times only) and 3 measurements were made to each sample to minimize errors in pipetting the viscous liquids. The above mixtures were then bleached in 365 nm ultraviolet light and the fluorescence intensity of the fluorophores remeasured.

Comparison of staining quality with different tissue clearing agents

All incubations were performed at 37°C unless otherwise specified. 1 mm-thick human motor cortex tissue slices were permeabilized in PBST at 37°C for 5 days, stained with anti-GFAP primary antibody at 1:100 dilution in PBST for 2 days, washed in PBST for 1 day, stained with AlexaFluor 568-conjugated anti-rabbit antibody at 1:100 dilution in PBST for 2 days, washed in PBST for 1 day, and incubated in various

tissue clearing formulae overnight at 37°C before finally proceeding to imaging. The tissue clearing formulae were prepared as described in the literature. Optimal imaging parameters were used for each sample for evaluating the staining qualities, namely background staining, nonspecific staining, and positive staining. The samples were then kept in their original refractive index homogenising solutions for 3 days and a second round of imaging was performed to evaluate the stability of staining. The imaging parameters were optimised for each sample, after which all imaging parameters were kept constant for each sample with no post-imaging contrast/brightness adjustments.
