# Supplementary material for: Next generation histology methods for three-dimensional imaging of fresh and archival human brain tissues
Source: Nat Commun. 2018 Mar 14;9:1066. doi: 10.1038/s41467-018-03359-w (PMC5852003; doi:10.1038/s41467-018-03359-w)
Supplement: Supplementary file 2 — Descriptions of Additional Supplementary Files [file 41467_2018_3359_MOESM2_ESM.pdf]

Descriptions of Additional Supplementary Files

File Name: Supplementary Movie 1
Description: Demonstration of the application of next generation histology protocols for three-dimensional visualization of human neural tissue architecture.
